# Integrated Multi‐Omics Analysis and Cross‐Model Validation Reveal Mitochondrial Signatures in Alzheimer's Disease

**DOI:** 10.1111/cns.70634

**Published:** 2025-10-27

**Authors:** Xuan Xu, Sha‐Sha Fan, Jiang Li, Hao Wu, Junwen He, Yang He, Xiang‐Yu Meng, Yin Shen

**Affiliations:** ^1^ School of Life Sciences Anhui Medical University Hefei Anhui China; ^2^ Health Science Center, Medical School Hubei Minzu University Enshi Hubei China; ^3^ Clinical Big Data Research Center, The Seventh Affiliated Hospital Sun Yat‐Sen University Shenzhen Guangdong China; ^4^ College of Informatics Huazhong Agricultural University Wuhan Hubei China; ^5^ School of Biomedical Engineering Anhui Medical University Hefei Anhui China

**Keywords:** Alzheimer's disease, biomarkers, machine learning, mitochondria, multiomics, polygenic risk scores

## Abstract

**Aims:**

Alzheimer's disease (AD) is a devastating neurodegenerative disorder where mitochondrial dysfunction is increasingly recognized as pivotal, yet its comprehensive molecular underpinnings remain incompletely understood. This study aimed to systematically identify and validate mitochondria‐related biomarkers associated with AD risk and brain resilience, thus elucidating the molecular mechanisms underpinning mitochondrial dysfunction in AD.

**Methods:**

We innovatively integrated a multi‐omics approach, encompassing genomics, DNA methylation, RNA‐sequencing, and miRNA profiles from the ROSMAP and ADNI cohorts (sample sizes ranging from 638 to 2090 per omic layer). Additionally, we applied 10 distinct machine learning methods to robustly identify and validate critical mitochondrial biomarkers relevant to AD progression. Subsequent validation was performed using a two‐tiered approach: an in vivo AD mouse model to establish phenotypic relevance and an in vitro H_2_O_2_‐induced oxidative stress model in HT22 cells to provide direct mechanistic validation.

**Results:**

Our computational analyses identified key biomarkers such as hsa‐miR‐129‐5p and *SLC6A12* as pivotal regulators and highlighted the importance of the tricarboxylic acid (TCA) cycle. Experimentally, our AD mouse model exhibited significant cognitive deficits and brain remodeling, linked to a specific transcriptomic signature. Our in vitro model functionally recapitulated mitochondrial dysfunction and oxidative stress. Crucially, a cross‐model analysis revealed a core signature of seven genes (including *APOE*, *CDKN1A*, and *CLOCK*) consistently dysregulated in both the cognitively impaired mouse brain and in neuronal cells subjected to direct oxidative insult. This provides powerful functional evidence linking our computationally derived targets, such as mitochondrial‐epistatic genes (*CLOCK*), to AD‐relevant pathology.

**Conclusion:**

These functionally validated findings provide deeper insights into the complex mitochondrial regulatory mechanisms involved in AD pathogenesis, offering robust biomarkers and novel potential avenues for developing targeted therapeutic strategies to address this challenging neurodegenerative disease.

## Graphical abstract

This study's framework begins with large‐scale data integration, leveraging the ROSMAP cohort for multi‐omics discovery (genotyping, DNA methylation, transcriptomics, miRNA) and the ADNI cohort for validation. This data feeds into a comprehensive analytical pipeline where differential expression analysis and an ensemble of 10 machine learning algorithms identify robust mitochondrial biomarkers of AD risk and brain resilience. To explore their biological context, a ceRNA network is constructed, and the key findings are subjected to a rigorous two‐tiered validation: in vivo confirmation of gene expression in an AD mouse model, followed by in vitro functional assessment of mitochondrial health (ROS, membrane potential) in an H_2_O_2_‐induced oxidative stress model in HT22 cells. This holistic discovery‐to‐validation workflow establishes a robust platform for identifying novel therapeutic targets and advancing our molecular understanding of AD.

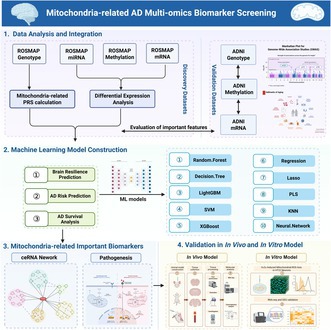

**Abbreviations**
AD: Alzheimer's disease
ADNI: Alzheimer's disease neuroimaging initiative
APP: amyloid precursor protein
AUC: area under the curve
Aβ: amyloid‐β
BBB: blood–brain barriers
ceRNA: competing endogenous RNA
CNS: central nervous system
DEG: differential expression gene
DMP: differentially methylated position
eQTL: expression quantitative trait loci
ETC: the electron transport chain
GBA: gut‐brain axis
GEO: gene expression omnibus
GLM: generalized linear model
GO: Gene ontology
GSEA: gene set enrichment analysis
HDL: high‐density lipoprotein
IBD: inflammatory bowel disease
KNN: K‐nearest neighbors
LDA: linear discriminant analysis
lncRNAs: long non‐coding RNAs
MCC: Markov cluster coefficient
MT: mitochondria
OXPHOS: oxidative phosphorylation
PRS: polygenic risk score
RF: random forest
ROC: receiver operating characteristic curve
ROS: reactive oxygen species
ROSMAP: Religious Orders Study and Memory and Aging Project
SVM: support vector machine

## 1 Introduction

Alzheimer's disease (AD) is characterized by a progressive clinical decline, with neuropathological features including extracellular amyloid‐β plaques and intracellular neurofibrillary tangles [1, 2, 3]. An expanding corpus of evidence points to a role for mitochondrial dysfunction in the pathogenesis of AD and may represent an early and crucial event [4, 5, 6]. Mitochondria are essential for maintaining cellular energy homeostasis, and their impairment is associated with increased oxidative stress, synaptic dysfunction, and neuronal death observed in AD [7]. However, the detailed molecular mechanisms underlying the relationship between mitochondria and AD remain to be fully elucidated [8].

There is a significant research gap regarding the complex interplay between mitochondrial dysfunction and the intricate genetic architecture of AD [9]. The heterogeneity of AD manifestations and the complex mitochondrial network make it challenging to pinpoint specific mechanisms and biomarkers [10]. Conventional statistical methodologies may be insufficient to characterize the subtle influence of mitochondrial dysfunction in AD, emphasizing the need for advanced analytical techniques.

Machine learning (ML) emerges as a promising avenue for dissecting the multifaceted nature of mitochondrial dysfunction in AD. ML algorithms are adept at modeling nonlinear relationships and interactions within large and complex datasets, such as multi‐omics data, which encompass genomics, transcriptomics, proteomics, and metabolomics [11]. By integrating these diverse data types, ML can identify AD‐related mitochondrial signatures that might otherwise be obscured [12, 13]. This approach could uncover novel mitochondrial biomarkers and provide insights into the pathophysiological mechanisms connecting mitochondrial dysfunction to AD progression. The application of ML to multi‐omics data is exceedingly pertinent in AD, considering the increasing acknowledgment of AD's heterogeneity and the burgeoning prospects of personalized medicine. ML's capacity to identify mitochondrial signatures indicative of AD risk or disease progression is a valuable asset. It holds the promise of enabling the development of targeted interventions and the customization of precision therapeutics, thereby personalizing care to match individual genetic profiles and clinical needs [14]. This method also improves our understanding of the variable manifestations of AD, offering insights into individual susceptibility and response to treatment.

**FIGURE 1:**
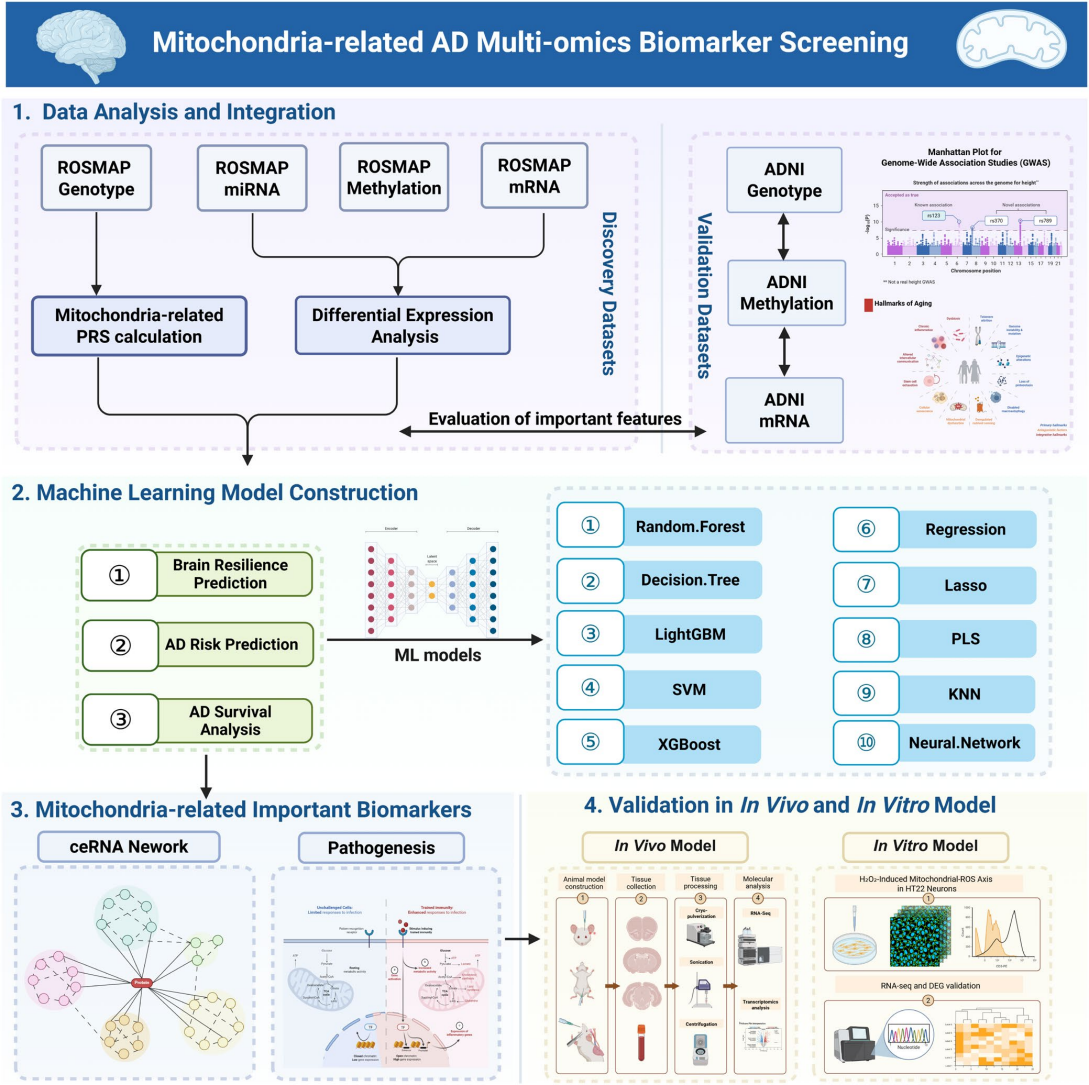
A multi‐pronged framework for the discovery and validation of mitochondrial‐related AD biomarkers through integrative multi‐omics analysis. The study begins with a large‐scale data integration phase, utilizing the ROSMAP cohort as the discovery dataset for four omics types (genotyping, DNA methylation, transcriptomics, miRNA) and the ADNI cohort for independent validation. This foundational data was then channeled into a comprehensive analytical pipeline, featuring differential expression analysis and an ensemble of 10 machine learning algorithms. The primary goal of this computational phase was to identify robust mitochondrial‐related biomarkers associated with AD risk and brain resilience. To elucidate the biological mechanisms underlying these biomarkers, we constructed a ceRNA regulatory network, revealing intricate mitochondrial‐associated pathways. To bridge the gap from computational prediction to biological reality, we initiated a two‐tiered validation process. First, the dysregulation of key gene signatures was confirmed in vivo in an AD mouse model via RNA‐Seq, establishing their relevance in a complex organismal context. Next, to specifically probe the predicted link to mitochondrial dysfunction at a cellular level, we established an in vitro oxidative stress model in HT22 hippocampal cells using H_2_O_2_. This allowed for the direct functional assessment of mitochondrial health through key indicators like reactive oxygen species (ROS) and membrane potential. This holistic approach—from human cohort data to animal models and finally to cellular assays—not only deepens our understanding of the AD molecular landscape but also provides a validated foundation for identifying novel therapeutic targets.

The innovation of our study lies in its comprehensive analysis, transcending the limitations of traditional single‐omics studies. By integrating diverse datasets and mitochondria‐related genetic risk scores, we aim to capture mitochondrial dysfunction in AD more accurately and identify crucial mitochondrial biomarkers that interact at the multi‐omics level. Our multi‐pronged framework employs a rigorous computational‐to‐experimental validation pipeline, beginning with large‐scale data integration from ROSMAP and ADNI cohorts across four omics types, followed by ensemble machine learning analysis to identify robust mitochondrial‐related biomarkers. To bridge the gap from computational prediction to biological reality, we implemented a two‐tiered validation strategy: first establishing phenotypic relevance through in vivo RNA‐Seq analysis in an AD mouse model, then providing direct mechanistic validation through in vitro oxidative stress experiments in HT22 hippocampal cells using H_2_O_2_‐induced mitochondrial dysfunction. In addition to enhancing our understanding of the molecular mechanisms underlying mitochondrial dysfunction in AD, our integrative methodology promotes the development of precision medicine strategies tailored to address mitochondrial pathology. This holistic approach—from human cohort data to animal models and finally to cellular assays—not only deepens our understanding of the AD molecular landscape but also provides a validated foundation for identifying novel therapeutic targets. The analytical and validation processes are outlined in Figure 1.

## 2 Methods

### 2.1 Study Subjects

The Religious Orders Study (ROS) and the Rush Memory and Aging Project (MAP) served as discovery datasets. Both studies involve older adults who undergo rigorous clinical evaluations and have consented to brain donation on death [15]. They have provided significant insights into the neuropathology of AD, the interrelation between risk factors and AD, and the clinical and imaging characteristics of MCI. Moreover, these studies have shed light on the complex interaction between neuropathological lesions and cognitive function in the aging process, as well as the possibility for resilient aging in the presence of considerable brain pathology. The results published here are in whole or in part based on data obtained from the AD Knowledge Portal (https://adknowledgeportal.org). For our investigation, the multi‐omics data from ROSMAP were utilized, encompassing genome‐wide genotypes (*n* = 2090), DNA methylation profiles (*n* = 740), RNA sequencing (*n* = 638), and MicroRNA (miRNA) profiles (*n* = 702).

The Alzheimer's Disease Neuroimaging Initiative (ADNI) datasets served as the validation dataset in our study. Established in 2003, ADNI, a public–private partnership, is dedicated to identifying the optimal clinical evaluations, imaging modalities, and biomarkers that will facilitate the development of therapeutic strategies for AD [16]. This collaborative effort has amassed a wealth of data on the mechanisms underlying the early preclinical and prodromal stages of AD, with comprehensive information accessible on their website (http://www.adni‐info.org/). For our validation, we curated corresponding data from ADNI that corresponded with the discovery dataset, encompassing genotyping data (*n* = 1550), methylation profiles (*n* = 1720), and RNA profiling (*n* = 811). We integrated genotyping data from three distinct ADNI phases: ADNI‐1, ADNI‐2, and ADNI‐GO. The clinical characteristics of the ROSMAP and ADNI samples are detailed in Table S1.

### 2.2 Data Processing

The processes for genotyping data and ADNI RNA expression were executed in accordance with the methodologies detailed in our recently released research [17, 18, 19]. The ROSMAP study encompassed 2090 genotyped samples, of which 1708 subjects were genotyped using the Affymetrix Gene Chip 6.0 (Affymetrix Inc., Santa Clara, CA, USA), and the remaining 382 subjects were genotyped using the Illumina HumanOmniExpress BeadChip (Illumina Inc., San Diego, CA, USA). Of the 1550 genotyped ADNI samples, 757 ADNI‐1 individuals were genotyped using the Illumina Human610‐Quad BeadChip (Illumina Inc., San Diego, CA, USA), while the remaining 793 ADNI‐GO/2 samples were genotyped using the HumanOmniExpress BeadChip (Illumina Inc., San Diego, CA, USA).

Methylation data in the ROSMAP study were derived from prefrontal cortex samples of 708 individuals, collected using the Illumina HumanMethylation450 BeadChip. These data underwent comprehensive quality control and were adjusted for confounding factors such as age, sex, and batch effects. The detailed methodologies and primary analyses are reported in De Jager et al. [20] Reprocessed RNA‐seq data from the ROSMAP cohort were sourced from the RNAseq harmonization study (syn9702085). The analytical workflow included key steps such as gene filtering, conditional quantile normalization, and the identification and adjustment of relevant covariates. Comprehensive methodologies and primary analyses are elucidated in Wan et al. [21] Expression profiles of approximately 700 miRNAs were procured from 734 frozen post‐mortem dorsolateral prefrontal cortex (DLPFC) samples, utilizing the NanoString nCounter miRNA expression assay. The dataset was preprocessed to include only miRNAs with a call rate of at least 95% and an absolute expression value above 15 in no less than 50% of the samples. Normalization was conducted using the variance stabilization normalization (VSN) method. Batch effects were mitigated using the ComBat algorithm, with cartridges specified as the batches. After pre‐processing, the dataset consisted of 309 miRNAs across 702 subjects.

In the ADNI study, the Illumina EPIC array (Illumina Inc., San Diego, CA, USA) was used to analyze 1720 samples derived from 653 individuals (categorized as cognitively normal (CN)/mild cognitive impairment (MCI)/AD), encompassing approximately 866,000 CpGs. A total of 200 samples were replicated across all chips, leading to the processing of 1920 samples in aggregate. When selecting participants, two experimental factors were taken into account: (1) time—indicative of the capacity to perform longitudinal studies (samples from patients with two or more visits); and (2) initial diagnosis and its progression over time (patients transitioning from CN to MCI, CN to AD, or MCI to AD). Given the study's focus on differential methylation analysis predicated on diagnosis, 199 duplicates and one triplicate detected on the EPIC array for technical replication purposes were incorporated but excluded from final analysis. Bristol‐Myers Squibb (BMS) offered gene expression profiles of 743 ADNI blood samples following quality control (QC) assessments. RNA quantity and quality were evaluated using NanoDrop and PerkinElmer Lab‐Chip GX instruments. Expression profiling was executed using the Affymetrix Human Genome U219 Array, comprising 530,467 probes that correspond to 49,293 transcripts. These probes were annotated with the R package “hgu219.db” aligned with the human genome GRCh37/hg19.

### 2.3 Differentially Expressed Analysis

Differential analyses of methylation, gene expression, and miRNA data were conducted utilizing the “limma” R package [22] (Linear Models for Microarray and RNA‐Seq Data). Initially, probes or transcripts that exhibited low expression levels or variability across samples were excluded to enhance statistical power and minimize noise interference. Methylation *β*‐values, serving as inputs, were normalized employing the normal‐exponential out‐of‐band (Noob) method from the “minfi” package, with batch effects adjustments made using the “ComBat” function within the “sva” package. Gene expression data underwent robust multi‐array average (RMA) method; subsequent log2 transformation of expression values was performed for analytical purposes. miRNA expression data were subjected to quantile normalization. A linear model was applied to each data type via the “limma” package, considering covariates such as age, sex, and experimental batch to mitigate potential confounding influences. The ROSMAP omics datasets were carefully stratified into three comparative cohorts corresponding to their respective phenotypes: AD versus CN, AD versus MCI, and MCI versus CN. Differentially methylated positions (DMPs), differentially expressed genes (DEGs), and differentially expressed miRNAs (DEMs) were detected through the computation of moderated t‐statistics, succeeded by the Benjamini‐Hochberg correction to regulate the false discovery rate (FDR). Ultimately, an appropriate adjusted *p* value threshold was selected based on the count of DMPs, DEGs, and DEMs.

To select significant features, we applied FDR thresholds tailored to the distinct statistical properties of each data type. For methylation and miRNA data, a more stringent threshold of FDR < 0.01 was used. These platforms typically assess a very large number of features where individual effect sizes can be subtle, necessitating a stricter cutoff to ensure the identification of high‐confidence signals. In contrast, for mRNA‐sequencing data, we used the standard and widely accepted threshold of FDR < 0.05. Transcriptomic data often exhibit a wider dynamic range and larger biological effect sizes, and this conventional threshold allows for the robust capture of biologically meaningful changes without being overly restrictive. This tailored approach is a common practice in multi‐omics analysis that accounts for the unique characteristics of each data modality.

### 2.4 Biological Functions Annotation

We selected appropriate methods for biofunctional annotation tailored to the unique characteristics of each data type. Following the identification of significant DMPs, the “missMethyl” R package was deployed to annotate these probes. The “getAnnotation” function from the “minfi” package furnished the necessary Illumina 450k annotation data, facilitating the mapping of each probe to its respective genomic location, gene context, and CpG island status. Furthermore, the “gometh” function was employed to perform a Gene Ontology (GO) enrichment analysis specifically adjusted for the number of probes per gene on the array, thereby reducing bias inherent in array‐based studies. This comprehensive annotation and pathway analysis provided insights into the biological significance and potential functional impact of the differentially methylated signatures.

DEGs were examined using the “clusterProfiler” R package [23]. GO and Kyoto Encyclopedia of Genes and Genomes (KEGG) pathway enrichment analyses were executed using the enrichGO and enrichKEGG functions, with significant terms identified at an FDR threshold of 0.05. Gene Set Enrichment Analysis (GSEA) was carried out using the gseGO and gseKEGG functions to identify enriched gene sets from the GO and KEGG databases. The “enrichplot” and “GseaVis” R packages were employed to visualize enrichment results. Bubble plots and tree plots offered an intuitive graphical representation of enriched terms, showcasing enrichment scores, gene ratios, and *p* values. “GseaVis” generated enrichment maps and dot plots to illustrate global patterns and leading‐edge subsets of enriched gene sets, thereby enhancing the interpretability of the results.

DEMs were annotated and analyzed using the miRNA Enrichment Analysis and Annotation Tool (miEAA) [24]. miEAA facilitates enrichment analysis across diverse categories, including GO terms, KEGG pathways, disease associations, and target gene enrichments. The analysis was performed using default settings, with the background reference defined as all detected miRNAs to ensure accurate enrichment results.

### 2.5 Mitochondrial Pathway Polygenic Risk Score Calculation

Polygenic Risk Scores (PRS) are used to quantify an individual's genetic predisposition to a specific trait or disease by aggregating the effects of multiple genetic variants. The computation of PRS begins with the selection of trait‐associated SNPs from genome‐wide association study (GWAS) summary statistics, succeeded by genotype data preparation and stringent quality control measures employing PLINK software (v1.90b4.10) [25]. Each SNP's risk allele count is weighted by its effect size derived from the GWAS, and the scores are summed to obtain the PRS for each individual. The formula for PRS is represented as:

$${\mathrm{PRS}}_{j}=\sum_{j=1}^{n}\beta_{j}\times {\text{Genotype}}_{\mathrm{ij}}$$

where ${\mathrm{PRS}}_{j}$ denotes the PRS for individual *i*. The summation is taken over *n* SNPs significantly associated with the trait of interest. $\beta_{j}$ signifies the effect size from the GWAS, reflecting the magnitude and direction of the association between the SNP and the trait. ${\text{Genotype}}_{\mathrm{ij}}$ represents the genotype of individual *i* at SNP *j*, coded as 0, 1, or 2, representing the number of risk alleles that the individual possesses for that SNP. The resultant PRS is standardized to facilitate comparability, and its predictive efficacy is ascertained through regression models within an independent cohort. PRSice‐2 [26] were utilized for the PRS computation and assessment. The pertinent QC procedures and calculation parameters are detailed in our preceding studies [17, 19].

PRS designed for specific mitochondria‐related biological pathways, termed MT pathway PRS, exploit genetic variants associated with particular disease mechanisms, facilitating the stratification of patient groups for targeted therapeutic interventions. The foundational data for the PRS analysis were derived from a recent AD GWAS study, encompassing 94,437 participants [27]. For our analysis, we culled summary statistics from 10,538,401 SNPs identified in stage 1, which included 21,982 patients and 41,944 controls. Our target files encompassed SNPs associated with mitochondrial genes, SNPs with epistatic interactions with mitochondrial SNPs significantly correlated with AD risk [19], reported AD risk loci identified from large‐scale genetic meta‐analyses [28], and mitochondrial pathway PRSs. The mitochondria‐associated pathways and their constituent genes were extracted from Human.MitoCarta3.0 [29] and PathCards [30]. To validate the accuracy of PRS calculations, pathways containing 10 or more genes were selected. Consequently, the PRSs for 74 mitochondrial pathways were constructed using processed genotype data from ROSMAP and ADNI.

### 2.6 Integrated Machine Learning for Biomarker Screening

For machine learning, differentially methylated positions (DMPs) and differentially expressed genes (DEGs) were selected as overlapping features from AD versus CN and AD versus MCI comparisons, using adjusted *p* value thresholds to prioritize consistent changes across disease stages, with a less stringent threshold for DEGs to account for larger effect sizes in mRNA data. Differentially expressed miRNAs (DEMs) were chosen from the AD versus CN comparison to capture the broadest AD‐relevant alterations, as AD versus MCI DEMs were a subset. Permutation importance was used to select top variables from the integrated “3omic” model (methylation, mRNA, miRNA), which were combined with top MT pathway PRSs to enhance predictive accuracy. The “tidymodels” package in R (https://tidymodels.tidymodels.org) provides a comprehensive suite of tools for constructing and evaluating machine learning models, rendering it exceptionally well suited for intricate predictive tasks. “Tidymodels” comprises a compilation of packages that work in concert to simplify the modeling workflow, including data preparation, model training, hyperparameter tuning, and performance evaluation. In this study, we harnessed the power of “tidymodels” to develop and appraise a diverse array of 10 distinct machine learning models, all aimed at forecasting AD risk and evaluating brain resilience. For each dataset, a meticulous hyperparameter optimization was performed employing a grid search strategy, with a grid size of 30 and 10‐fold cross‐validation (10‐CV) resampling. This stringent tuning regimen guaranteed that the model exhibiting the utmost predictive proficiency was chosen for finalization. The synergistic application of various “tidymodels” constituent packages, including “rsample” for data partitioning, “recipes” for data preprocessing, “parsnip” for model specification, and “yardstick” for performance metrics, provided a robust framework for the analysis. The models that factored into our analysis were methodically divided into four principal categories: tree‐based models, linear models, support vector machines, and other models.

#### 2.6.1 Tree‐Based Models

(1) Random Forest: An ensemble learning technique that crafts multiple decision trees during the training phase, culminating in a consensus decision, typically the class mode, for classification tasks.

(2) Decision Tree: A model that recursively partitions the data based on feature values, thereby creating a hierarchical tree structure representative of decision‐making processes.

(3) LightGBM: A high‐performance gradient boosting framework underpinned by tree‐based learning algorithms, noted for its efficiency and speed.

(4) XGBoost: An optimized gradient boosting library recognized for its high efficiency, adaptability, and robustness in handling large‐scale data.

#### 2.6.2 Linear Models

(5) Logistic Regression: A statistical model predicting the likelihood of a binary outcome, contingent upon various predictor variables; Linear Regression: A foundational regression analysis method that delineates the relationship between a dependent variable and an array of independent variables.

(6) Least Absolute Shrinkage and Selection Operator (Lasso): A regression method that concurrently performs variable selection and regularization, thereby potentially enhancing the precision of predictions.

(7) Partial Least Squares (PLS): A statistical technique for establishing fundamental linear relationships between predictor and response variables, particularly advantageous in regression scenarios with multicollinear data.

#### 2.6.3 Support Vector Machine

(8) Support Vector Machine (SVM): A supervised learning algorithm that identifies the optimal hyperplane, aiming to maximize the margin separation between disparate classes.

#### 2.6.4 Other Models

(9) Neural Network (NN): A computational model drawing inspiration from neural networks in the human brain, adept at discerning intricate patterns within datasets through layered interconnected nodes.

(10) K‐Nearest Neighbors (KNN): An instance‐based learning algorithm that assigns classifications to new instances based on a majority vote among their k‐nearest neighbors within the training dataset.

For binary classification tasks, such as distinguishing AD from non‐AD cases, we employed the area under the receiver operating characteristic curve (AUC) and accuracy (ACC) as evaluation metrics. Furthermore, leveraging five brain resilience metrics grounded in genetic risk measures from our preceding research [17], we constructed regression models to assess brain resilience as a continuous variable. For this purpose, we utilized root mean square error (RMSE) and the coefficient of determination (RSQ) as our metrics for model performance evaluation.

### 2.7 Interpretation of the Importance of Model Variables

Each model was individually ranked according to each metric, and the aggregate of these rankings was employed to ascertain the final model hierarchy. For each predictive factor (one categorical metric and five regression metrics), we selected variables that appeared in the top three models with the highest combined rankings for either AUC and ACC in classification tasks, or RMSE and RSQ in regression tasks. For each of the six predictive metrics, the top three models with the highest predictive efficiency were selected for subsequent variable importance analysis. For the interpretation of these selected models, we employed the “DALEX” [31] and “DALEXtra” R packages. Specifically, to determine variable importance, we utilized the permutation‐based variable importance method available in “DALEX.” This technique calculates the drop in model performance when the values of a single predictor are randomly shuffled, with a larger performance decrease indicating greater importance. We computed these importance scores for every variable within each of the top three models for a given task. To derive a single, robust list of key predictors, we then aggregated these results by calculating the average rank and average permutation‐based importance score for each variable across the three models. The top 20 variables with the best aggregated scores were selected as the final set of critical features, ensuring their consistent predictive power across multiple high‐performing algorithms. For the Random Forest model, we further used the “vivid” R package to visualize not only individual feature importance but also to uncover significant interaction effects between variables, thereby enhancing the biological interpretability of our findings.

### 2.8 Survival Analysis of Principal Variables

Based on the cumulative variable importance scores from the top three models, the most critical top 50% of variables were selected for subsequent analysis. These variables underwent survival analysis using various ADNI omics datasets with the “survival” and “survminer” R packages. Due to the unavailability of miRNA expression data in the ADNI dataset, we resorted to employing miRNA target genes as predicted by “multiMiR,” opting for the highest‐scoring target genes that were validated through luciferase assays. For each variable, Cox proportional hazards models were fitted meticulously to assess their individual impact on the risk of AD, with the estimation of hazard ratios (HRs) serving to quantify the relative risk attributed to each variable. The analytical process encompassed the curation of pertinent omics datasets, the application of Cox models to estimate HRs, and the graphical representation of outcomes using Kaplan–Meier curves and forest plots facilitated by the “ggplot2” package. This holistic methodology allowed for the quantification of each feature's contribution to the risk of AD, yielding robust insights into the pivotal prognostic factors that regulate the onset and progression of the disease.

### 2.9 ceRNA Network Construction

#### 2.9.1 Prediction of Gene Targeting Important miRNAs

To predict important (HR *p* value < 0.05) miRNA target genes, the “multiMiR” [32] R package was utilized, which amalgamates data from several databases, including miRTarBase [33], TargetScan [34], and miRDB [35]. Interactions with high confidence, as evidenced by support from multiple databases and robust experimental validation, were selected and integrated with the DEGs analysis. This methodology enabled the precise identification of biologically relevant miRNA‐target interactions, thereby offering a comprehensive understanding of miRNA regulatory roles.

#### 2.9.2 Prediction of miRNAs Regulating Important Genes

Similarly, genes and methylation sites with significant HR (*p* value < 0.05) were deemed risk‐significant. For these elements, regulatory miRNAs were predicted, with a preference for those documented in both the TarBase and miRTarBase databases, facilitating the construction of subsequent ceRNA networks.

#### 2.9.3 Prediction of miRNA–lncRNA Interaction

The interactions between miRNAs and lncRNAs were predicted using the starBase database [36] (https://rnasysu.com/encori/). The important miRNAs identified in the previous steps were cross‐referenced with this database, which compiles interaction data from CLIP‐Seq and degradome sequencing experiments. By prioritizing interactions with the strongest experimental support, potential regulatory relationships were scrutinized and identified.

#### 2.9.4 Prediction of miRNA–circRNA Interaction

The interaction prediction between circRNAs and miRNAs was conducted using CircBank (http://www.circbank.cn/) [37], a machine learning‐based tool specializing in the identification of miRNA binding sites on circRNAs. CircRNA sequences were sourced from public databases or literature. Utilizing the CircBank API, these sequences were input to predict binding sites for miRNAs of interest. The predicted interactions underwent analysis to pinpoint potential binding sites on circRNAs, complete with associated confidence scores. The circRNA exhibiting the highest confidence level was selected as the final prediction.

#### 2.9.5 Visualization of ceRNA Networks

For the visualization of ceRNA networks, Cytoscape (v3.10.0) was engaged. Interaction data encompassing miRNAs, lncRNAs, circRNAs, and mRNAs were collated and imported into Cytoscape, which procedurally constructed the network. Nodes represented ceRNAs, and edges symbolized their interactions. Cytoscape's layout algorithms were harnessed to arrange and customize the network for enhanced clarity, with node and edge properties adjusted to emphasize particular interactions. Plugins “cytoHubba” and “ClueGO” were additionally employed for network structural analysis and functional annotation, thereby providing deeper insights.

### 2.10 Functional Annotation of Candidate SNPs

The Functional Mapping and Annotation of GWAS (FUMA) [38], an integrative tool encompassing 18 biological databases and resources, was employed to conduct a comprehensive annotation of the subset of single‐nucleotide polymorphisms (SNPs) with the highest modeled importance in both the ROSMAP and ADNI studies. Utilizing FUMA's SNP2GENE function, each SNP was mapped to its potential target genes through positional mapping, expression quantitative trait loci (eQTL) mapping, and chromatin interaction mapping. Subsequently, the GENE2FUNC function was applied for gene annotation and functional analysis. Gene expression patterns across various tissues were scrutinized using Genotype‐Tissue Expression (GTEx) data, and heat maps were crafted to graphically represent the average log2 (RPKM) values. Differentially expressed gene (DEG) sets across tissues were identified, and enrichment analysis was performed to evaluate their participation in biological pathways and functional categories, employing annotations from Reactome [39], MSigDB [40], and WikiPathways [41] annotations. Corrections for multiple testing were implemented using the Bonferroni method, with a significance threshold established at an adjusted *p* value of less than 0.05, particularly for chromatin interaction pathways and GO biological processes. The analysis leveraged Ensembl v92 and GTEx v8 datasets, with a focus on 13 distinct brain tissues to clarify the implications of brain‐related SNPs.

### 2.11 Animal Experiments

Specific pathogen‐free (SPF) grade 10‐week‐old male Kunming mice (Sibeck Biotechnology, Henan, China) were used to establish an induced model of subacute aging and dementia mimicking AD. The mice were randomly assigned to the model group and the age‐matched control group, with five animals each. For the model group, the mice were subcutaneously injected with D‐galactose (Aladdin Biochemical Technology Co. Ltd., Shanghai, China) at a dosage of 120 mg/kg/day for induction of subacute aging and sodium nitrite (Aladdin Biochemical Technology Co. Ltd., Shanghai, China) at a dosage of 55 mg/kg/day for the enhancement of cognitive impairment. The injections were administered daily for a continuous period of 60 days after a 7‐day acclimation. During the same period, the age‐matched control group received daily subcutaneous injections of an equivalent volume of sterile saline. This combined treatment was designed to induce brain aging, cognitive impairment, and simulate dementia‐like symptoms in the mice, allowing for a direct comparison against a non‐induced, age‐matched baseline. During the experimental period, the mice were maintained under standard laboratory conditions, with free access to food and water, and monitored regularly for health and behavioral changes.

After modeling treatment, the mice were subjected to behavioral assessment using the Morris‐Water Maze (MWM), similarly as previously described [42]. Briefly, the mice were first trained for 5 days to assess their learning ability (e.g., the training stage) and then tested for memory ability with the platform removed after a 1‐day break (e.g., the testing stage). For the training experiments, the time taken to reach the platform was recorded each day, averaging the performance across three quadrants. For the testing experiments, the latency to locate and the times crossing the platform region were recorded.

The animals were subjected to euthanasia after MWM experiments. The hippocampus was retrieved similarly as previously described. Hippocampus RNA was extracted and subjected to RNA‐seq for 3 animals in the model and control groups. RNA‐seq data analysis was performed similarly as previously described [42]. qPCR for the neuron synapse marker *Grin2a* and the microglia inflammation marker *Tnfrsf11b*, using the following primers:

*Grin2a*:

F: ACGTGACAGAACGCGAACTT.

R:TCAGTGCGGTTCATCAATAACG.

*Tnfrsf11b*:

F: CCTTGCCCTGACCACTCTTAT.

R: CACACACTCGGTTGTGGGT.

*Gapdh*:

F: GGTTGTCTCCTGCGACTTCA.

R: TGGTCCAGGGTTTCTTACTCC.

All the animal experiments were ethically approved by the Institutional Review Board of Hubei Minzu University.

### 2.12 Assessment of Oxidative Stress and Mitochondrial Integrity

The murine hippocampal cell line, HT22, was maintained in Dulbecco's Modified Eagle's Medium (DMEM) supplemented with 10% fetal bovine serum (FBS) and 1% penicillin/streptomycin. All cell culture was conducted at 37°C in a humidified atmosphere containing 5% CO_2_. Key reagents included a Reactive Oxygen Species Assay Kit (DCFH‐DA; Biosharp, Cat. No. BL714A), Mito‐Tracker Red CMXRos (Beyotime, Cat. No. C1999S), Trypsin (KeyGEN BioTECH, Cat. No. KGL2102), and 20 mm glass‐bottom confocal dishes (Biosharp, Cat. No. BS‐20‐GJM). Quantitative analysis was performed using a cytoStellar AX flow cytometer (Challenbio), and cellular imaging was conducted on a Nikon A1 confocal laser scanning microscope (Nikon Corporation).

To quantify intracellular reactive oxygen species (ROS), HT22 cells were seeded into 24‐well plates at a density of 1.6 × 10^5^ cells/mL in a volume of 500 μL per well. Following an initial 24‐h incubation period to allow for cell attachment and growth to 70%–80% confluency, the culture medium was replaced with fresh medium containing hydrogen peroxide (H_2_O_2_) at a final working concentration of 500 μM. The cells were then incubated for an additional 24 h to induce oxidative stress. Post‐treatment, the medium was aspirated, and the cells were gently rinsed once with 1× Dulbecco's phosphate‐buffered saline (DPBS). Cells were subsequently dissociated with trypsin, collected by centrifugation, and washed twice with 1× DPBS. The resulting cell pellet was resuspended in 100 μL of a 10 μM working solution of 2′,7′‐dichlorofluorescin diacetate (DCFH‐DA) and incubated for 30 min at 37°C. Following this incubation, cells were washed twice with 1 mL of 1× DPBS to remove any non‐internalized DCFH‐DA probe. Finally, the cells were resuspended in 1× DPBS to a final concentration of 1 × 10^6^ cells/mL for immediate analysis by flow cytometry. ROS‐associated fluorescence was detected using an excitation wavelength of 488 nm and an emission wavelength of 525 nm. The acquired data were analyzed to determine both the percentage of ROS‐positive cells and the mean fluorescence intensity (MFI) as a measure of the overall intracellular ROS level.

For the assessment of mitochondrial membrane potential (MMP), HT22 cells were seeded into 20 mm glass‐bottom confocal dishes at a density of 1 × 10^5^ cells/mL in a volume of 800 μL per dish. The cells were cultured and subsequently treated with 500 μM H_2_O_2_ for 24 h, following the same protocol described for the ROS assay. After the treatment period, the medium was aspirated, and the cells were washed twice with 1× DPBS. A dual‐staining working solution was prepared by diluting 1 μL of 1000× Mito‐Tracker Red CMXRos stock and 1 μL of 1000× Hoechst 33342 stock into 998 μL of Assay Buffer. Each dish was then incubated with 300 μL of this staining solution for 30 min at 37°C, protected from light. Following incubation, the staining solution was removed, and the cells were gently rinsed twice with 1× DPBS before adding 300 μL of fresh Assay Buffer for imaging. Fluorescent images were acquired using the confocal microscope. Mito‐Tracker Red CMXRos fluorescence, indicative of mitochondrial membrane potential, was captured using an excitation wavelength of 579 nm and an emission wavelength of 599 nm, while nuclei stained with Hoechst 33342 were visualized using an excitation wavelength of 350 nm and an emission wavelength of 461 nm.

### 2.13 Transcriptome Profiling of Murine Hippocampal HT22 Cells

Total RNA was extracted from HT22 cells, representing three biological replicates per experimental group, using TRIzol reagent (Thermo Fisher Scientific) according to the manufacturer's protocol. Briefly, cells cultured to 80%–90% confluency in 6‐well plates were lysed directly in 1 mL of TRIzol. Following phase separation with chloroform, the aqueous phase containing RNA was collected, and RNA was precipitated with isopropanol. The resulting RNA pellets were washed with 75% ethanol and resuspended in RNase‐free water. The concentration and purity of the extracted RNA were assessed using a NanoDrop 2000 spectrophotometer, while RNA integrity was evaluated using an Agilent 5300 Bioanalyzer, with samples having an RNA integrity number (RIN) > 6.5 deemed suitable for library construction.

Sequencing libraries were prepared from 1 μg of total RNA per sample. Messenger RNA (mRNA) was enriched from total RNA using oligo(dT) magnetic beads and subsequently fragmented into approximately 300 bp segments. These fragments were used as templates for first‐strand cDNA synthesis with random hexamer primers, followed by second‐strand cDNA synthesis. The resulting double‐stranded cDNA underwent end‐repair, A‐tailing, and ligation to sequencing adapters. The adapter‐ligated fragments were then amplified by PCR to generate the final cDNA libraries.

The constructed libraries were quantified using a Qubit 4.0 fluorometer and pooled in equimolar ratios. The pooled libraries were subjected to cluster generation on a cBot system, followed by 150 bp paired‐end sequencing on a NovaSeq X Plus platform (Illumina).

For bioinformatic analysis, raw sequencing reads were first processed using fastp to remove adapter sequences, low‐quality reads (quality score < 20), and reads with a high percentage of unknown bases (*N* > 10%), yielding high‐quality clean data. These clean reads were then aligned to the mouse reference genome (ENSEMBL GRCm39) using the STAR aligner in two‐pass mode. Gene expression levels were quantified from the aligned reads using featureCounts from the Subread package, which generated a matrix of read counts per gene for all samples. This count matrix served as the input for subsequent differential expression analysis.

## 3 Results

### 3.1 Differential Expression Analysis Identifies Key AD‐Associated Omics Features

A rigorous differential expression analysis comparing AD, mild cognitive impairment (MCI), and cognitively normal (CN) individuals has discerned substantial alterations in methylation, mRNA, and miRNA profiles. Utilizing significance thresholds tailored to each data type (FDR < 0.01 for methylation/miRNA and < 0.05 for mRNA to account for differing statistical properties), we identified distinct molecular signatures. Within the methylation dataset, 19,713 and 128 differentially methylated positions (DMPs) were found in the AD versus CN and AD versus MCI comparisons, respectively (adj. *p* < 0.01). Notably, 117 DMPs were observed to overlap between these two comparative groups. In contrast, the MCI versus CN comparison showed less pronounced differences, with 2699 DMPs identified (*p* < 0.01; Figure S1A–C, Table S2). Examination of the mRNA dataset uncovered 4710 and 49 differentially expressed genes (DEGs) in the AD versus CN and AD versus MCI comparisons, respectively (adj. *p* < 0.05), sharing 49 DEGs in common. Again, the MCI versus CN comparison exhibited less significant changes, identifying only 128 DMPs (*p* < 0.01), with 119 of these appearing in the AD versus CN comparison, and 3 present in all three groups (*CCDC69*, *SLC6A12*, and *SLC6A9*; Figure S2A–C, Table S2). The miRNA dataset exposed 29 and six differentially expressed miRNAs (DEMs) in the AD versus CN and AD versus MCI comparisons, respectively (adj.*p* < 0.01), with the six DEMs from the AD versus MCI comparison being a subset of those found in the AD versus CN comparison. Similarly, in the MCI versus CN comparison, the differences were less pronounced, revealing three DEMs (*p* < 0.01), with 2 of them (hsa‐miR‐95 and hsa‐miR‐375) appearing in the AD versus CN comparison as well (hsa‐miR‐95, hsa‐miR‐375; Figure S3A–C, Table S2). The alterations observed in the MCI versus CN comparison, though generally less numerous than in the AD versus CN group, represent the earliest detectable molecular signatures of cognitive decline. However, to identify the most robust biomarkers associated with the definitive disease state and the critical molecular switches involved in disease progression, our subsequent in‐depth analyses, including the development of machine learning models, primarily focused on the features identified from the AD versus CN comparison (representing the strongest pathological signal) and the AD versus MCI comparison (representing the transition to severe disease). This targeted approach ensures the selection of features with the highest potential for diagnostic and prognostic relevance.

### 3.2 Functional Enrichment Analysis Highlights Mitochondrial Processes in AD

Functional enrichment analysis of the differentially expressed features highlighted the involvement of mitochondrial dysfunction and metabolic alterations in AD pathogenesis. In the comparison between AD and CN, differentially methylated sites were significantly enriched in morphogenesis (GO:0009653, adj. *p* = 1.33 × 10^−28^) and organ development (GO:0007275, adj. *p* = 1.45 × 10^−24^), and metabolic pathways, including neuroactive ligand‐receptor interactions (hsa04080, adj. *p* = 1.41 × 10^−6^) and calcium signaling pathways (hsa04020, adj. *p* = 8.42 × 10^−6^; Figures S1D,E and S4, Table S3).

The mRNA data from the AD versus CN group demonstrated significant enrichment in synaptic signaling, membrane potential regulation, and neurotransmitter secretion. Pathways associated with cognitive functions such as learning and memory were notably enriched. The majority of the Kyoto Encyclopedia of Genes and Genomes (KEGG) pathway results were attributed to the apelin signaling pathway, circadian rhythm, and calcium signaling. In the AD versus MCI group, numerous mitochondria‐related functions were enriched, such as mitochondrial respiratory chain complex assembly (GO:0033108, adj. *p* = 0.00075). Cellular localization showed marked enrichment in mitochondrial protein‐containing complexes (GO:0098798, *p* = 0.0016) and the mitochondrial inner membrane (GO:0005743, *p* = 0.00015; Figures S2 and S5, Table S3).

For the miRNA data, the AD versus CN group showed significant enrichment of the insulin receptor signaling pathway (GO:0008286, adj. *p* = 2.14 × 10^−8^) and DNA‐methyltransferase activity (GO:0009008, adj. *p* = 3.08 × 10^−8^). Among the three comparison groups, the predicted target genes with the highest numbers were *HSPA1B* (mitochondrial localization gene), *CDK6* (mitochondrial epistasis gene), and *IGF1R* (mitochondrial epistasis gene; Figure S3D–L, Table S3).

### 3.3 Mitochondria‐Related Polygenic Risk Scores Identify Critical Pathways in AD

**FIGURE 2:**
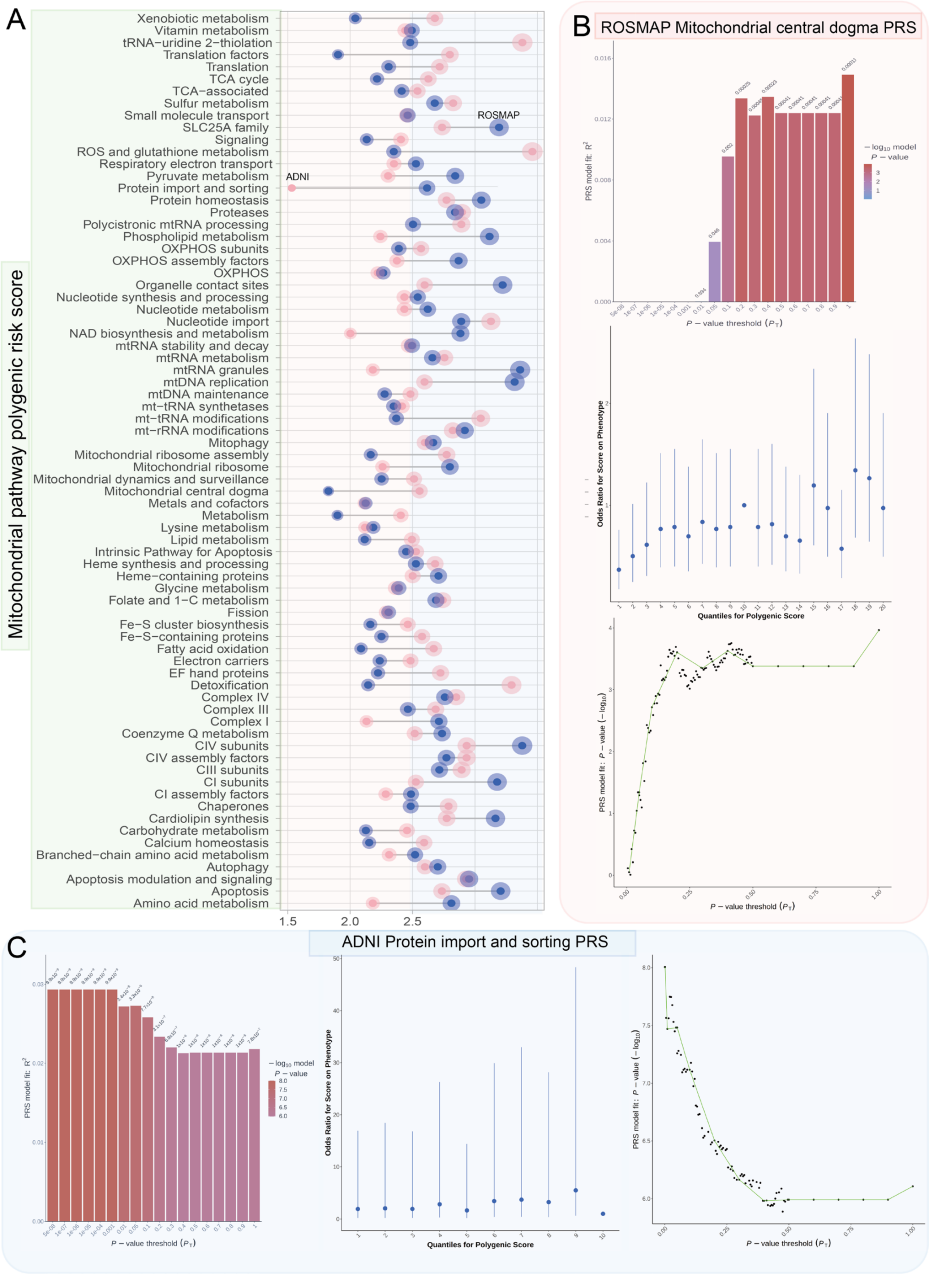
Mitochondrial pathway‐specific polygenic risk scores reveal consistent associations with AD risk across the ROSMAP and ADNI cohorts. (A) Polygenic Risk Scores (PRS) tailored for mitochondrial‐related biological pathways (MT pathway PRS) leverage genetic variants linked to specific disease mechanisms, enabling the stratification of patient cohorts for targeted therapeutic interventions. By focusing on genetic risk factors associated with mitochondrial function, these specialized PRS models offer a powerful tool for elucidating the intricate genetic architecture of AD and identifying high‐risk individuals who may benefit from pathway‐specific interventions. (B) In our comprehensive analysis of 74 mitochondria‐related pathways, the PRS model exhibiting the most robust performance on the ROSMAP dataset was derived from the Mitochondrial Central Dogma pathway. This model exhibited a considerable substantial R‐squared (*R* ^2^) value of 0.029 with a highly significant *p* value of 9.87 × 10^−9^, which highlights the critical role of this pathway in the genetic risk for AD in the ROSMAP cohort. (C) When assessed on the ADNI dataset, the PRS model with the most notable performance was based on the Protein Import and Sorting pathway, with an *R* ^2^ of 0.015 and a significant *p* value of 0.00011. This finding indicates that genetic variants associated with mitochondrial protein import and sorting contribute significantly to AD risk in the ADNI cohort. Notably, a convergence was observed among the top 20 best‐performing MT Pathway PRS models across both datasets, with six pathways consistently standing out: Lysine Metabolism, General Metabolism, Metals and Cofactors, Mitochondrial Ribosome, OXPHOS Assembly Factors, and Signaling. This overlap highlights the shared genetic risk factors and biological mechanisms underlying AD pathogenesis in both cohorts. The MT Pathway PRS specific to the Metals and Cofactors pathway demonstrated the most compelling combined performance in both databases, with *R* ^2^ values of 0.0075 (*p* = 0.0059) in ROSMAP and 0.0077 (*p* = 0.0034) in ADNI. This consistent association across different cohorts underscores the significant role of metal homeostasis and cofactor metabolism in the genetic risk for AD. Furthermore, the Small Molecule Transport pathway‐specific PRS exhibited the most analogous model performance in both datasets, with *R* ^2^ values of 0.0035 (*p* = 0.061) in ROSMAP and 0.0035 (*p* = 0.05) in ADNI, suggesting that genetic variants related to mitochondrial small molecule transport may confer similar risk for AD across different populations. In contrast, the mtRNA Granules pathway‐specific PRS exhibited the most disparate model performance, with an *R* ^2^ of 0.00043 (*p* = 0.51) in ROSMAP and 0.0066 (*p* = 0.0092) in ADNI. This suggests a potentially more nuanced role of this pathway in the context of AD (Table S8). This discrepancy underscores the heterogeneity in the genetic architecture of AD and the necessity of considering cohort‐specific factors when interpreting genetic risk.

To assess genetic risk associated with AD, we developed PRS models using 74 mitochondria‐related pathways (MT pathway PRSs), carefully selected to represent key mitochondrial processes implicated in AD pathogenesis. These pathways, derived from comprehensive databases such as Human.MitoCarta3.0 [29] and PathCards [30], were chosen to include only those with ten or more genes to ensure robust PRS calculations. The PRS model derived from the Mitochondrial Central Dogma and Protein Import and Sorting pathway demonstrated superior performance in both the Religious Orders Study and Memory and Aging Project (ROSMAP) cohort (*R* ^2^ = 0.029, *p* = 9.87 × 10^−9^) and the ADNI cohort (*R* ^2^ = 0.015, *p* = 0.00011; Figure 2). Six pathways were consistently identified among the top 20 best‐performing models across both datasets. These pathways included Lysine Metabolism, General Metabolism, Metals and Cofactors, Mitochondrial Ribosome, OXPHOS Assembly Factors, and Signaling. The Metals and Cofactors pathway‐specific MT Pathway PRS exhibited the most notable combined performance in analyses from both databases (ROSMAP: *R* ^2^ = 0.0075, *p* = 0.0059; ADNI: *R* ^2^ = 0.0077, *p* = 0.0034). Furthermore, the Small Molecule Transport pathway‐specific PRS model displayed the most similar performance in both cohorts (ROSMAP: *R* ^2^ = 0.0035, *p* = 0.061; ADNI: *R* ^2^ = 0.0035, *p* = 0.05), while the mtRNA Granules pathway‐specific PRS model showed the most divergent performance (ROSMAP: *R* ^2^ = 0.00043, *p* = 0.51; ADNI: *R* ^2^ = 0.0066, *p* = 0.0092; Table S4).

### 3.4 Integrative Multi‐Omics Machine Learning Models Improve AD Risk Prediction

**FIGURE 3:**
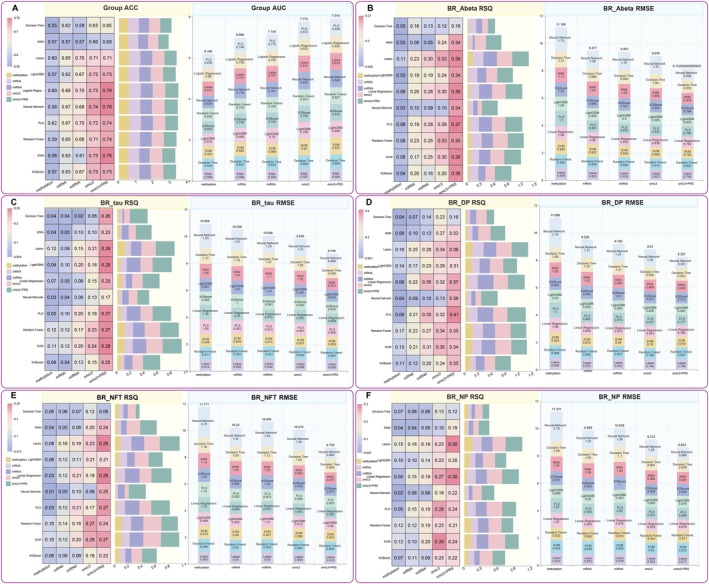
Integrative multi‐omics prediction model based on machine learning algorithms enhances the predictive performance for AD and brain resilience indicators. This figure illustrates the performance of ten distinct machine learning algorithms across five feature sets: Single‐omics (methylation, mRNA, miRNA), integrated multi‐omics (3omic), and multi‐omics with Polygenic Risk Scores (3omic + PRS). Heatmaps represent the performance of all models, while bar plots highlight the results of the top‐performing model for each feature set. (A) Performance of classification models for predicting AD status. The left heatmap shows model accuracy (ACC), while the right bar plot displays the area under the curve (AUC). A clear trend of improved performance is observed as data integration progresses. The integrated 3omic + PRS model, specifically when using a neural network, achieved the highest performance, with an ACC of 0.76 and an AUC of 0.846. (B–F) Performance of regression models for predicting five distinct brain resilience indicators. For each panel, the left heatmap presents the *R*‐squared (RSQ) value (higher values indicate better performance), while the right bar plot shows the root mean square error (RMSE) (lower values indicate better performance) for the top model of each feature set. (B) Prediction of resilience to Aβ pathology (BR_Abeta). The most significant improvement was observed with the 3omic + PRS feature set, where partial least squares (PLS) regression achieved the highest RSQ of 0.37 and the lowest RMSE of 0.786. (C) Prediction of resilience to tau pathology (BR_tau). The 3omic + PRS model again demonstrated superior performance, with support vector machine (SVM) achieving the highest RSQ of 0.28 and PLS yielding the lowest RMSE of 0.856. (D) Prediction of resilience to diffuse plaques (BR_DP). The PLS model using the 3omic + PRS feature set yielded the best performance, with an RSQ of 0.41 and an RMSE of 0.777. (E) Prediction of resilience to neurofibrillary tangles (BR_NFT). For this indicator, the linear regression model with 3omic + PRS features outperformed other models, achieving an RSQ of 0.29 and an RMSE of 0.832. (F) Prediction of resilience to neuritic plaques (BR_NP). The Lasso model with 3omic + PRS features provided the most accurate predictions, with an RSQ of 0.30 and an RMSE of 0.842. Collectively, these results consistently demonstrate a stepwise improvement in predictive power: The integrated multi‐omics model (3omic) outperforms any single‐omic approach, and the further incorporation of mitochondria‐related genetic risk scores (3omic + PRS) provides the highest accuracy across all tasks. By leveraging single‐omics data, we developed classification models to distinguish AD from non‐AD individuals, utilizing 117 DMPs, 49 DEGs, 29 DEMs, and 74 MT pathway PRSs. While the top‐performing algorithm varies by task, the neural network frequently exhibits robust performance, particularly with the most complex feature sets.

To develop robust predictive models for AD risk, we constructed classification and regression models using single‐omics data, selecting 117 DMPs, 49 DEGs, 29 DEMs, and 74 mitochondria‐related polygenic risk scores (MT pathway PRSs). These features were chosen based on their significance in differential expression analyses and relevance to mitochondrial dysfunction in AD. Specifically, the 117 DMPs were the overlapping features identified in both AD versus CN and AD versus MCI comparisons, ensuring robust methylation changes across disease stages. The 49 DEGs were selected as the overlapping genes from AD versus CN and AD versus MCI comparisons, capturing consistent gene expression alterations. The 29 DEMs were derived from the AD versus CN comparison, as the 6 DEMs in AD versus MCI were a subset, ensuring the inclusion of the broadest miRNA alterations relevant to AD. The 74 MT pathway PRSs were constructed from curated mitochondria‐related pathways, as described above (Figure 2, Table S4). The integration of these three molecular features—methylation data (methy), mRNA expression data (mRNA), and miRNA expression data (miRNA), into a single model (termed “3omic”), resulted in a marked improvement in predictive performance compared to models that relied solely on a single type of omic features (Figure 3). Using permutation importance to rank features based on their contribution to model performance (e.g., reduction in RMSE or increase in *R* ^2^), we identified the top 20 variables from the 3omic model, considering the importance of features across the three omics datasets. These were then combined with the top 20 MT pathway PRS metrics (Figure S6, Table S5). The incorporation of these genetic risk factors related to mitochondria further enhanced the models' predictive accuracy. Remarkably, the most significant improvements were observed in genetic indicators of brain resilience, especially those based on Amyloid‐beta (Abeta) (Neural Network: RMSE_max↓_ = 0.924, RSQ_max↑_ = 0.245) and tau profiles (Neural Network: RMSE_max↓_ = 0.941, Decision Tree: RSQ_max↑_ = 0.177). Among the models, the neural network (nnet) exhibited the most substantial enhancement in predictive performance (Table S5).

### 3.5 Feature Screening and Validation Elucidate Relationships With AD Risk

**FIGURE 4:**
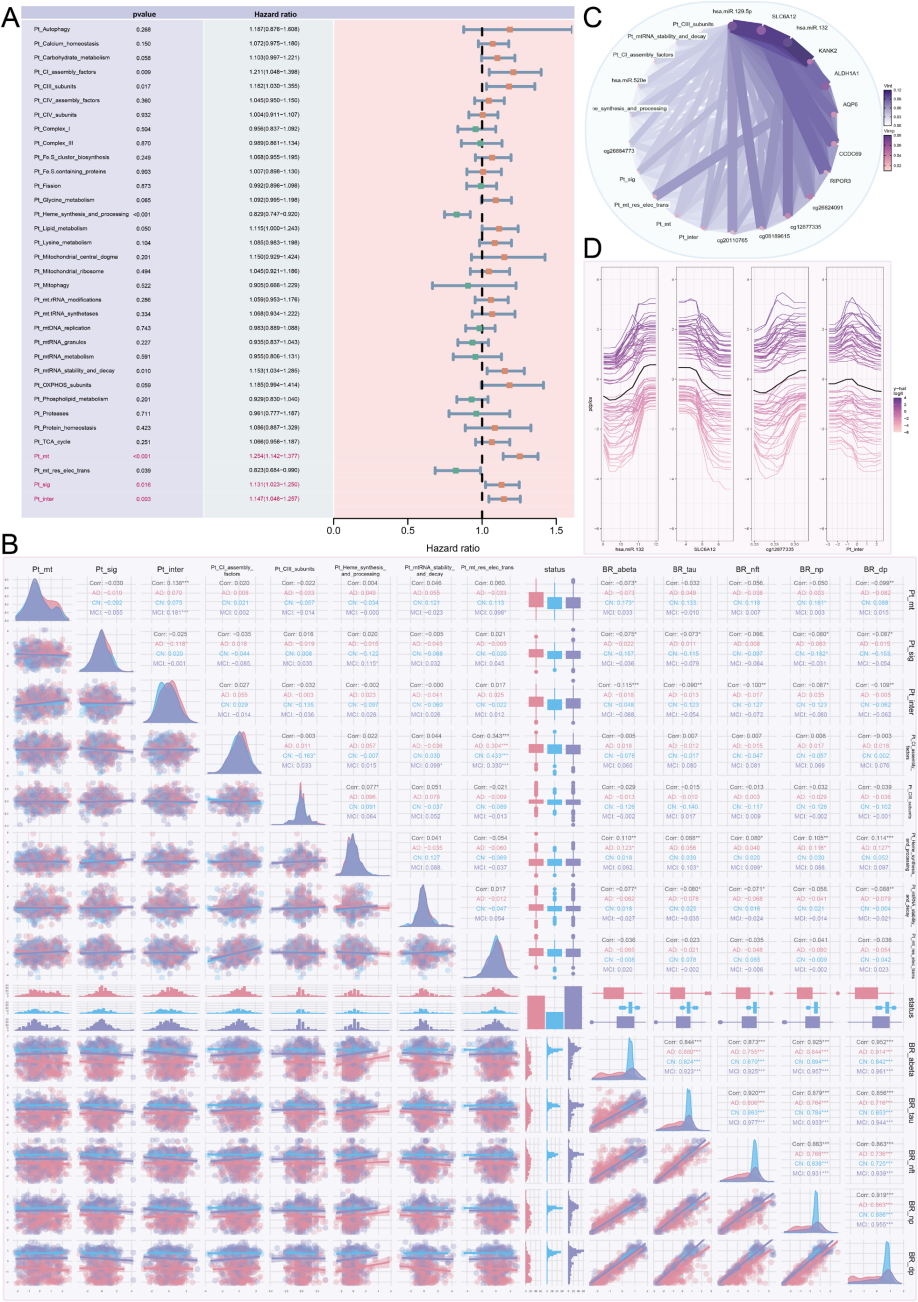
Integrative analysis reveals complex interactions among mitochondrial genetic risk factors and their association with AD phenotypes in the ROSMAP cohort. (A) Forest plot of multivariate Cox regression analysis for mitochondrial pathway PRSs. This plot displays the hazard ratios (HR) for AD risk associated with various mitochondrial pathway Polygenic Risk Scores (PRSs). Of the pathways tested, eight were significantly associated with AD risk (*p* < 0.05). PRSs for mitochondrial‐localized genes (Pt_mt), mitochondrial‐epistasis genes (Pt_inter), AD risk factor genes (Pt_sig), Complex I assembly factors (Pt_CI_assembly_factors), Complex III subunits (Pt_CIII_subunits), and mitochondrial RNA stability and decay (Pt_mtRNA_stability_and_decay) were all associated with an increased risk of AD (HR > 1). Conversely, PRSs for Heme synthesis and processing and Respiratory electron transport (Pt_mt_res_elec_trans) were found to be significantly protective (HR < 1). (B) Correlation matrix of significant PRSs, AD status, and brain resilience metrics. This plot visualizes the interrelationships between key variables, with density plots on the diagonal, scatter plots in the lower triangle, and Pearson correlation coefficients in the upper triangle. A significant positive correlation was observed between Pt_inter and Pt_mt (Overall Corr = 0.138, *p* < 0.001), an association that was strongest in the MCI group (MCI Corr = 0.223). Notably, a brain resilience metric based on Aβ levels (BR_abeta) showed a significant negative correlation with Pt_inter (Overall Corr = −0.115, *p* < 0.001), indicating that higher genetic risk in this pathway corresponds to lower resilience to amyloid pathology. Additionally, a strong positive correlation was found between the PRSs for Pt_mt_res_elec_trans and Pt_CI_assembly_factors (Overall Corr = 0.343, *p* < 0.001), reflecting their biological linkage. (C) Chord diagram illustrating interactions from a random forest model. This diagram visualizes the interaction strengths among the top significant multi‐omics features in predicting AD status. The width and color intensity of the chords correspond to the strength of the interaction. The analysis revealed strong interactions between features from different omic layers, including miRNAs (hsa‐miR‐129‐5p, hsa‐miR‐132), genes (*SLC6A12*, *ALDH1A1*, *KANK2*, *RIPOR3*), methylation sites (cg26884773), and genetic risk scores (Pt_inter, Pt_sig), highlighting a complex, interconnected regulatory network driving AD risk. (D) Partial Dependence (PDP) and Individual Conditional Expectation (ICE) plots. These plots show the marginal effect of the top individual features on the predicted AD risk from the random forest model. The thick black line represents the average prediction (PDP), while the thin colored lines show predictions for individual subjects (ICE). The plots visualize the direction and magnitude of each feature's influence. For example, increasing levels of *SLC6A12* are associated with a decreased AD risk, while increasing levels of Pt_inter are associated with an increased risk, consistent with findings from the Cox regression. Similarly, higher expression of hsa‐miR‐132 is linked to lower AD risk.

**FIGURE 5:**
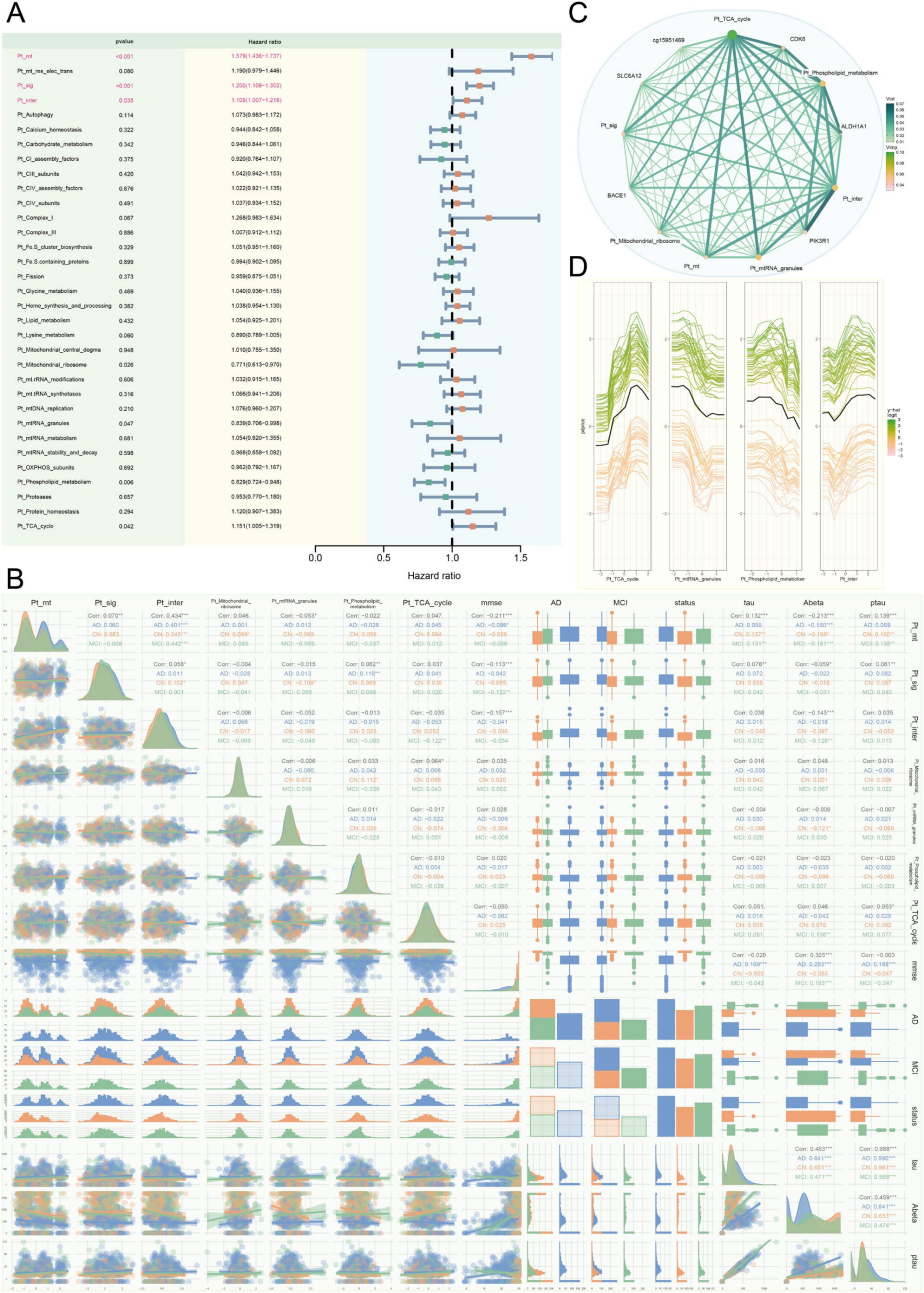
Integrative analysis uncovers significant associations between mitochondrial genetic risk factors and AD phenotypes in the ADNI cohort. (A) Forest plot of multivariate Cox regression analysis for mitochondrial pathway PRSs. This plot displays the hazard ratios (HR) and confidence intervals for AD risk. In the ADNI cohort, six MT pathway PRSs demonstrated a significant association with AD risk (*p* < 0.05). Consistent with the ROSMAP cohort, PRSs derived from mitochondrial‐localized genes (Pt_mt, HR = 1.436), mitochondrial‐epistasis genes (Pt_inter, HR = 1.109), and GWAS‐significant genes (Pt_sig, HR = 1.100) were all positively associated with AD risk. Furthermore, PRSs for Pt_Mitochondrial_ribosome, Pt_Phospholipid_metabolism, and Pt_TCA_cycle also showed significant impacts. Notably, Pt_mt emerged as the most substantially significant variable for impacting AD risk across both datasets (*p*_*ROSMAP*_ = 2.21 × 10^−6^, *p*_*ADNI*_ = 4.19 × 10^−21^). (B) Correlation matrix of significant PRSs, cognitive scores, and CSF biomarkers. This panel visualizes complex correlations, with density plots on the diagonal and pairwise correlation coefficients in the upper triangle. Cognitive function, measured by MMSE scores, demonstrated significant negative correlations with the major PRSs: Pt_mt (Corr = −0.221, *p* < 0.001), Pt_inter (Corr = −0.157, *p* < 0.001), and Pt_sig (Corr = −0.113, *p* < 0.001), indicating that higher mitochondrial genetic risk is tightly linked to worse cognitive status. Furthermore, Pt_mt significantly correlated with all three classical CSF biomarkers: Abeta (negative correlation, Corr = −0.213, *p* < 0.001), tau (positive correlation, Corr = 0.132, *p* < 0.001), and Ptau (positive correlation, Corr = 0.189, *p* < 0.001), underscoring its relevance to core AD pathology. A positive correlation was also noted between Pt_Phospholipid_metabolism and Pt_sig (Corr = 0.082, *p* < 0.01). (C) Chord diagram illustrating variable interactions from a random forest model. This plot displays the strong synergistic relationships among the top multi‐omics features in the ADNI dataset. Key interaction clusters were identified, including those linking the genetic metabolism PRSs (e.g., Pt_TCA_cycle and Pt_Phospholipid_metabolism) with the mitochondrial epistatic gene CDK6 and other significant genes like ALDH1A1. Another strong cluster connected Pt_TCA_cycle, the general mitochondrial PRS (Pt_mt), and the Pt_mtRNA_granules PRS. The strongest interactions involved Pt_inter and the gene PIK3R1, highlighting complex regulation between mitochondrial genetic risk and cellular signaling components. (D) Partial Dependence and Individual Conditional Expectation plots. These plots illustrate the marginal effect of the four most critical features on the predicted model outcome (y‐hat score). The black line represents the average predicted trend (PDP). For all four features shown (Pt_TCA_cycle, Pt_mtRNA_granules, Pt_Phospholipid_metabolism, and Pt_inter), increasing values of the PRS generally correspond to higher predicted risk scores, reinforcing their pivotal predictive role established in the model importance rankings.

ROSMAP data demonstrated that all four omics features effectively distinguished AD risk groups, with a more early onset in individuals exhibiting higher expressions (*p* < 0.0001; Figure S7). In ADNI, MT pathway PRSs and miRNA‐targeted genes (Table S6) most significantly predicted AD risk (*p* < 0.0001), with methylation and mRNA profiles also showing significant discriminatory power (*p* = 0.031 and 0.022; Figure S8). Multivariate Cox regression analysis was employed to prioritize key AD risk features (*p* < 0.05). In MT pathway PRSs, eight were significant in ROSMAP (Figure 4A) and seven in ADNI (Figure 5A), converging in three categories: PRSs derived from mitochondrial‐localized genes (Pt_mt), PRSs associated with mitochondrial‐epistasis genes (Pt_inter) in our previous [17], and PRSs of genes identified as AD risk factors in large‐scale GWAS meta‐analysis (Pt_sig) [27]. Pt_mt emerged as the variable with the strongest significance for the impact of AD risk in both datasets (*p*_*ROSMAP*_ = 2.21 × 10^−6^, *p*_*ADNI*_ = 4.19 × 10^−21^). All were significantly positively associated with AD risk (HR_max_ = 1.579).

In ROSMAP, five methylation sites (cg12877335, cg26824091, cg26884773, cg20110765, cg08189615) showed a significant AD risk association, compared to one in ADNI (cg15951469). mRNA data revealed six significant features in ROSMAP and two in ADNI, with *ALDH1A1* and *SLC6A12* negatively correlating with AD risk in both. Notably, *SLC6A1*2, uniquely identified as a DEG across all three comparison groups (AD versus CN, AD versus MCI, and MCI versus CN), exhibited a significant negative correlation with AD risk in both cohorts (HR_ROSMAP_ = 0.784, HR_ADNI_ = 0.673). The miRNA data from ROSMAP identified three significant miRNAs (hsa‐miR‐129‐5p, hsa‐miR‐520e, and hsa‐miR‐132), whereas ADNI identified five potential miRNAs (hsa‐miR‐23b‐3p, hsa‐miR‐23b‐5p, hsa‐miR‐29a‐3p, hsa‐miR‐132‐3p, and hsa‐miR‐885‐5p) after target gene conversion. Notably, hsa‐miR‐132 was the sole miRNA validated in both datasets (Figures S7 and S8).

### 3.6 Variable Correlation Analysis Unveils Interconnections Among Multi‐Omics Characteristics

Following multivariate Cox regression, we analyzed significant variable correlations in ROSMAP and ADNI. Pt_inter and Pt_mt showed strong positive correlations in both datasets (Corr_ROSMAP_ = 0.138, Corr_ADNI_ = 0.434, *p* < 0.001), particularly pronounced in MCI. Brain resilience, as indicated by Abeta levels, demonstrated the strongest negative correlation with Pt_inter (Corr = −0.115, *p* < 0.001). Additionally, Pt_mt_res_elec_trans (Respiratory electron transport pathway PRS) correlated positively with Pt_CI_assembly_factors (CI assembly factors pathway PRS; Corr = 0.343, *p* < 0.001; Figure 4A,B). In ADNI, Mini‐Mental State Examination (MMSE) scores were negatively correlated with Pt_mt, Pt_inter, and Pt_sig (Corr = −0.221, *p* < 0.001), Pt_inter (Corr = −0.157, *p* < 0.001), and Pt_sig (Corr = −0.113, *p* < 0.001). Cerebrospinal fluid (CSF) biomarkers, including Abeta (Corr = −0.213, *p* < 0.001), tau (Corr = 0.132, *p* < 0.001), and Ptau (Corr = 0.189, *p* < 0.001), significantly correlated with Pt_mt (Figure 5A,B).

In ROSMAP, *RIPOR3* and *SLC6A12* displayed the strongest positive correlation (Corr = 0.777, *p* < 0.001), whereas hsa‐miR‐132 exhibited the highest positive association with brain resilience metrics, and *SLC6A12* showed the strongest negative correlation. In ADNI, the strongest positive correlation was observed between *PIK3R1* and *CDK6* (Corr = 0.531, *p* < 0.001), and *BACE1* demonstrated the most significant negative correlations with CSF indices (Corr_CSF‐tau_ = −0.251, Corr_CSF‐ptau_ = −0.262, *p* < 0.001; Corr_CSF‐Abeta_ = −0.227, *p* < 0.01; Figures S9 and S10). Random forest modeling in ROSMAP revealed strong interactions between multiple variables, including hsa‐miR‐129‐5p, hsa‐miR‐132, *SLC6A12*, and *KANK2* (Figure 4C), while in ADNI, significant interactions were observed among Pt_TCA_cycle, Pt_Phospholipid_metabolism, *CDK6*, *ALDH1A1*, Pt_TCA_cycle, Pt_mt, Pt_mtRNA_granules, and Pt_inter with *PIK3R1* (Figure 5C). The bias‐dependence plot illustrated the substantial effect of important features on AD status (Figures 4D and 5D).

### 3.7 ceRNA Network Analysis Reveals Key Regulatory Interactions and Molecular Hubs

**FIGURE 6:**
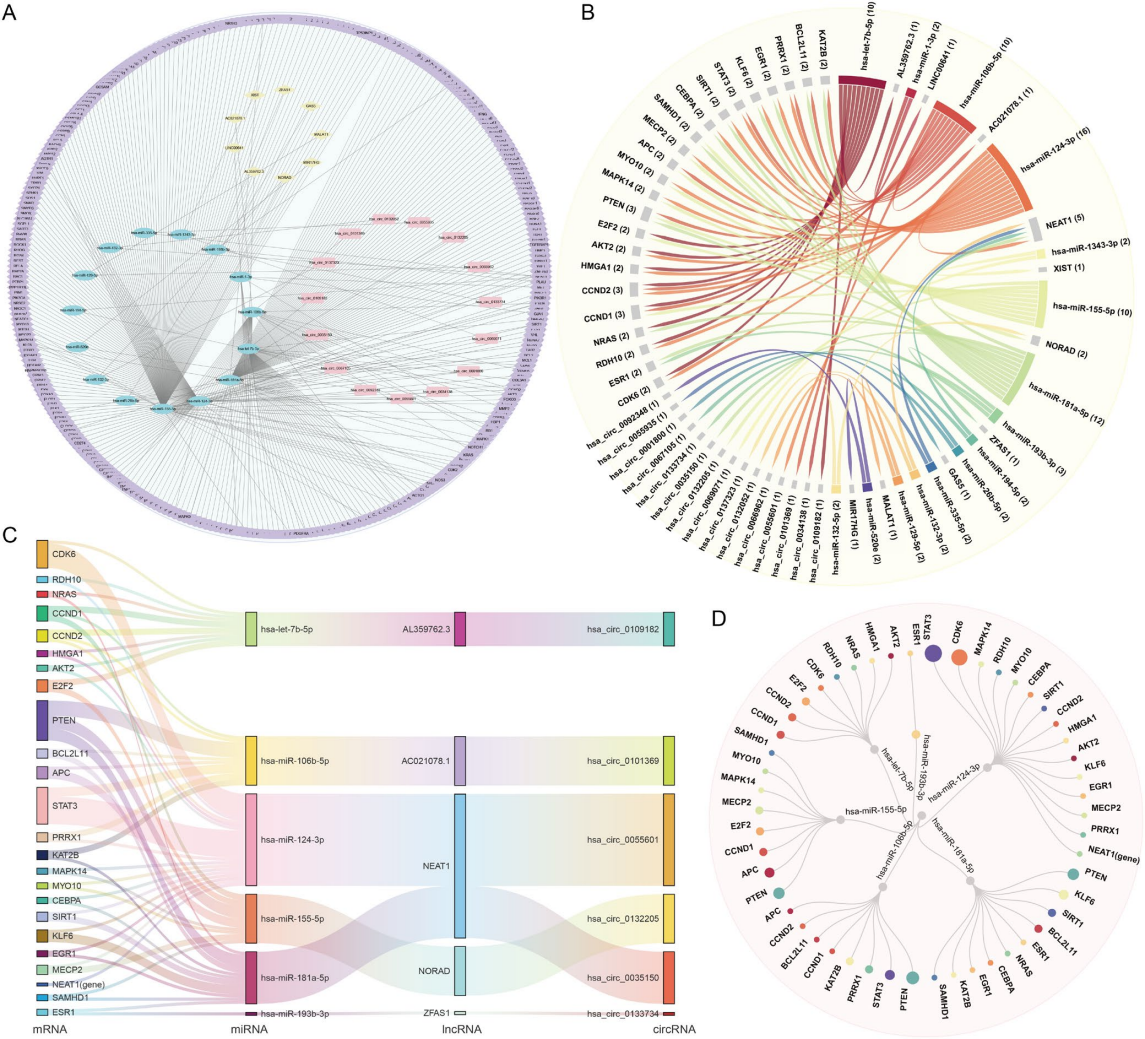
Integrative analysis of the ceRNA network reveals key regulatory hubs and interactions in AD pathogenesis. (A) Global view of the constructed competing endogenous RNA (ceRNA) network. This comprehensive network was constructed to elucidate the complex regulatory interactions among pivotal molecular features, integrating 15 key miRNAs (cyan nodes), 10 lncRNAs (yellow nodes), 15 circRNAs (red nodes), and their 305 target genes (peripheral nodes). The edges represent the 330 predicted miRNA‐gene regulatory associations, providing a complete overview of the cross‐talk between coding and non‐coding RNAs implicated in AD. (B) Chord diagram highlighting connectivity of central network hubs. This plot visualizes the interactions between the most connected genes (left) and non‐coding RNAs (right). The number in parentheses indicates the degree of connectivity (i.e., number of interactions). Network topology analysis identified *hsa‐miR‐124‐3p* as a top regulatory hub, a finding supported by its high connectivity (targeting 16 genes) in this diagram. Similarly, the lncRNA NEAT1 emerges as a critical sponge, interacting with five different miRNAs. (C) Sankey diagrams illustrating specific ceRNA regulatory axes. These diagrams detail the flow of regulation within key sponge networks, showing how lncRNAs and circRNAs can sequester miRNAs to modulate the expression of their target mRNAs. A prominent example shows how *hsa‐miR‐124‐3p* regulates key mitochondrial epistatic genes, including *STAT3* and *CDK6*. The diagram visualizes how this interaction is modulated by the lncRNA *NEAT1* and the circRNA *hsa_circ_0055601*, which act as sponges for hsa‐miR‐124‐3p. (D) Hub‐and‐spoke diagram of key miRNA regulators and their targets. This view highlights the pleiotropic effects of the most central miRNAs identified through network analysis. At the gene and lncRNA levels, PTEN and NEAT1 (shown in panel C) emerged as hubs with the richest interaction profiles. This diagram illustrates this by showing that a gene hub like PTEN is targeted by multiple central miRNAs (e.g., hsa‐miR‐155‐5p). This visualization underscores the multifaceted regulatory roles of these molecules within the broader ceRNA network. Interactive visualization files are available at: https://github.com/XuanXu1230/ML‐AD/tree/main/networkplot/.

A competing endogenous RNA (ceRNA) network analysis was conducted to delineate the regulatory interactions among pivotal elements, integrating 15 miRNAs, 305 genes, 15 circRNAs, and 10 long non‐coding RNAs (lncRNAs) and 330 miRNA‐gene regulatory associations (comprising 305 genes; Figure 6A, Table S7). The miRNA hsa‐miR‐124‐3p exhibited the highest number of associations in all nodes with Markov cluster coefficient (MCC) greater than or equal to two (Figure 6B). This miRNA established the most robust connections with mitochondrial epistasis genes such as *STAT3* and *CDK6* (mitochondrial epistasis genes; Figure 6C). Furthermore, *PTEN* and *NEAT1* were identified as hubs with the highest diversity of interactions at the gene and lncRNA levels (Figure 6D).

### 3.8 Annotation of Critical SNPs Elucidates AD Pathological Mechanism

Genetic analysis identified two PRS collections comprising 620 SNPs, mostly intronic (Figure S11A). Annotation pinpointed two risk regions on chromosome 19 (19:45149613–45382034 and 19:45706952–45709881; Figure S9B) and four lead SNPs (rs714948, rs57537848, rs6859, rs620807). Brain expression quantitative trait loci (eQTL) analysis indicated these SNPs' influence on *CEACAM19* expression across brain tissues (Table S8). Hi‐C data (GSE87112) disclosed interactions between rs56261258 (*PVR*) and genes such as ENSG00000216588 (*IGSF23*), ENSG00000159915 (*ZNF233*), and ENSG00000062370 *(ZNF112)* (Figure S11C). Comparative analysis of 68 mapped genes revealed significant expression trends for *CLPTM1*, *MARK4*, and *PVRL2*, etc., across 54 GTEx tissues (Figure S11D, Tables S8 and S9). Notably, 22 zinc finger protein family genes, including *ZNF45*, *ZNF221*, and *ZNF155*, were enriched in RNA Polymerase II transcription (adj. *p* = 1.45 × 10^−6^) and lipid metabolism pathways (adj.*p* = 3.67 × 10^−5^). GWAS catalog annotation showed significant enrichment in AD/age‐related processes, such as AD or high‐density lipoprotein (HDL) levels (adj. *p* = 6.69 × 10^−15^), body mass index age interaction (adj. *p* = 3.75 × 10^−6^) and cerebrospinal fluid AD pathology levels (Aβ1‐42, p‐tau, t‐tau, etc.; adj. *p* = 8.77 × 10^−5^/8.70 × 10^−3^/1.15 × 10^−2^; Figure S11E, Table S10).

### 3.9 An AD Mouse Model Exhibits Cognitive Deficits and Brain Remodeling Associated With a Specific Transcriptomic Signature

**FIGURE 7:**
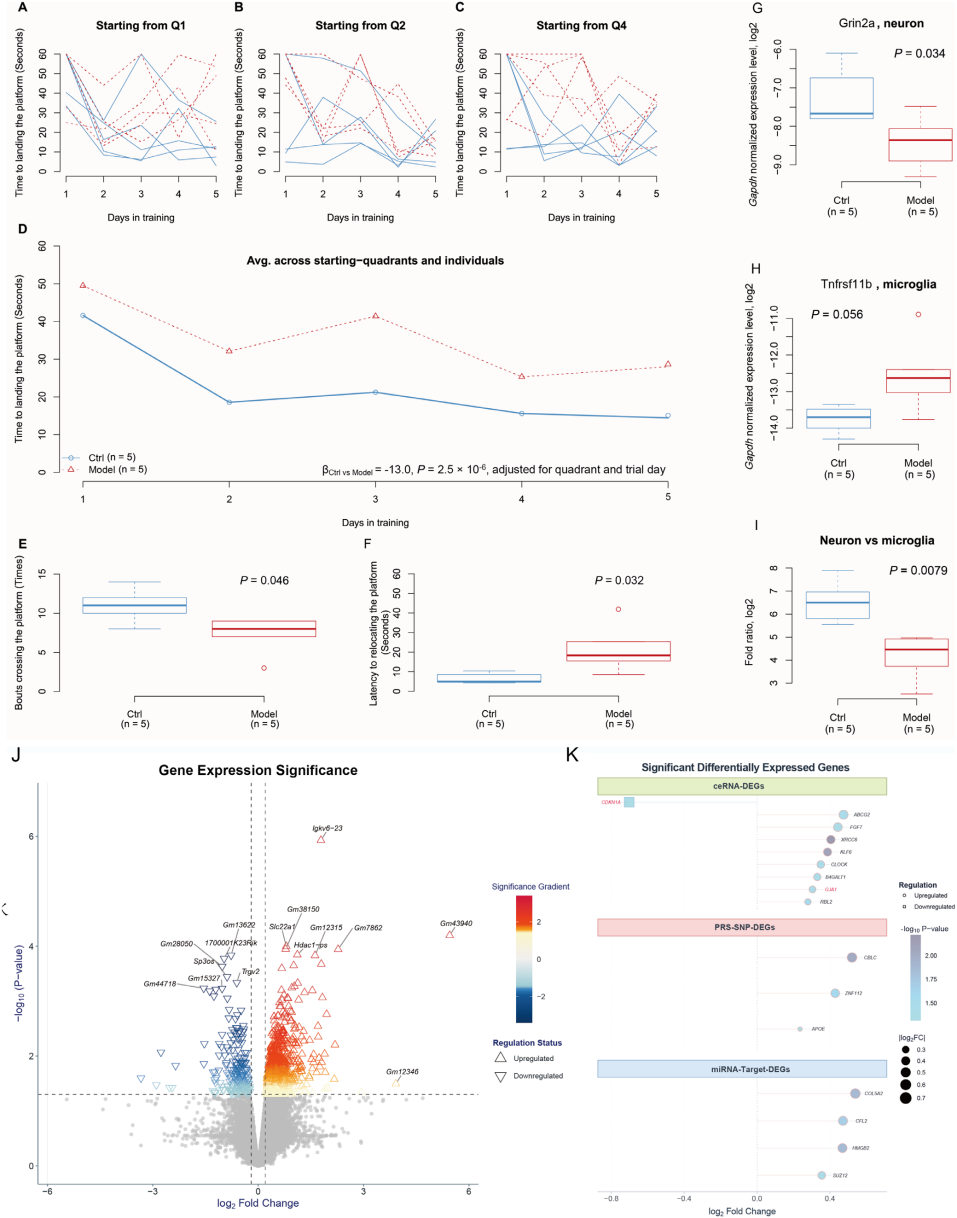
Cognitive impairment and brain remodeling in D‐galactose–induced aging mice and RNA‐seq analyses. (A–F) Morris Water Maze (MWM) assays demonstrate significant learning and memory deficits in the AD model mice. (A–C) Individual learning curves for control (Ctrl, blue solid lines) and AD model (Model, red dashed lines) mice during the 5‐day training phase, starting from different quadrants (Q1, Q2, Q4). Control mice consistently show a decrease in the time required to find the platform, whereas model mice exhibit erratic and impaired learning. (D) The average learning curve across all individuals and starting quadrants. The model mice (red triangles) consistently took significantly longer to find the platform compared to control mice (blue circles), demonstrating a profound learning deficit (Linear regression βCtrl vs. Model = −13.0, *p* = 2.5 × 10^−6^). (E, F) Spatial memory assessment during the probe test phase (platform removed). (E) Model mice crossed the former platform location significantly fewer times than control mice (*p* = 0.046). (F) Model mice also exhibited significantly longer latency to first reach the platform's previous location (*p* = 0.032). Both results indicate impaired spatial memory retention. (G–I) qPCR analysis provides molecular evidence of adverse brain remodeling. (G) The expression of the neuronal marker Grin2a was significantly downregulated in the brains of model mice (*p* = 0.034), suggesting neuronal dysfunction or loss. (H) Conversely, the expression of the microglia marker Tnfrsf11b showed a trend towards upregulation in model mice (*p* = 0.056), indicative of neuroinflammation or gliosis. (I) Consequently, the calculated Neuron versus microglia ratio was significantly decreased in the model group (*p* = 0.0079), providing strong evidence for a shift from a healthy neuronal environment towards a pro‐inflammatory state. (J, K) RNA‐seq analysis validates the differential expression of key genes identified in human multi‐omics data. (J) A volcano plot illustrating the results of the differential gene expression analysis between model and control mice. A total of 549 significantly differentially expressed genes (DEGs) were identified, indicating a widespread transcriptional response in the AD model. Upregulated genes are shown as red triangles, and downregulated as blue inverted triangles. (K) This dot plot highlights the significant DEGs that were previously prioritized by our computational analyses. Key genes from the ceRNA network (e.g., upregulated CDKN1A, KLF6; downregulated GJA1), genes linked to PRS‐derived SNPs (e.g., upregulated CBLC, ZNF112), and key miRNA target genes (e.g., upregulated SUZ12; downregulated CFL2) all showed significant differential expression in the mouse model, providing strong in vivo validation for their relevance to AD pathogenesis.

To establish a relevant in vivo model for neurodegeneration, we first evaluated the cognitive and neuropathological changes in mice treated with D‐galactose and sodium nitrite. Behavioral testing using the Morris Water Maze (MWM) revealed significant cognitive impairment in the AD model mice (*n* = 5) compared to controls (*n* = 5). Specifically, the model mice showed a markedly reduced learning ability, as indicated by a longer time required to find the hidden platform across training days (*β*_Ctrl vs. Model_ = −13.0, linear regression, *p* = 2.5 × 10^−6^; Figure 7A–D). Their long‐term spatial memory was also compromised, evidenced by fewer platform crossings (median = 11 vs. 8, *p*_Wilcoxon_ = 0.046; Figure 7E) and a longer latency to first reach the platform location during the probe trial (median = 4.9 vs. 18.3 s, *p*_Wilcoxon_ = 0.032; Figure 7F). At the molecular level, qPCR analysis of brain tissue demonstrated adverse brain remodeling. We observed a significantly decreased ratio of the neuronal marker *Gri2a* to the microglia marker *Tnfrsfr11b* (log2 fold ratio, median = 6.5 vs. 4.5, *p* = 0.0079; Figure 7G–I), a signature consistent with neuronal loss and neuroinflammation seen in aging brains and AD [42, 43, 44]. To identify the molecular landscape underlying this phenotype, we performed RNA‐seq on brain tissue, identifying 549 significantly differentially expressed genes (DEGs) (Figure 7J and Table S11). This comprehensive characterization established a robust animal model with clear behavioral and molecular phenotypes, providing a transcriptomic signature directly associated with cognitive decline.

### 3.10 An in Vitro Model Functionally Links Oxidative Stress to Mitochondrial Dysfunction in Neuronal Cells

**FIGURE 8:**
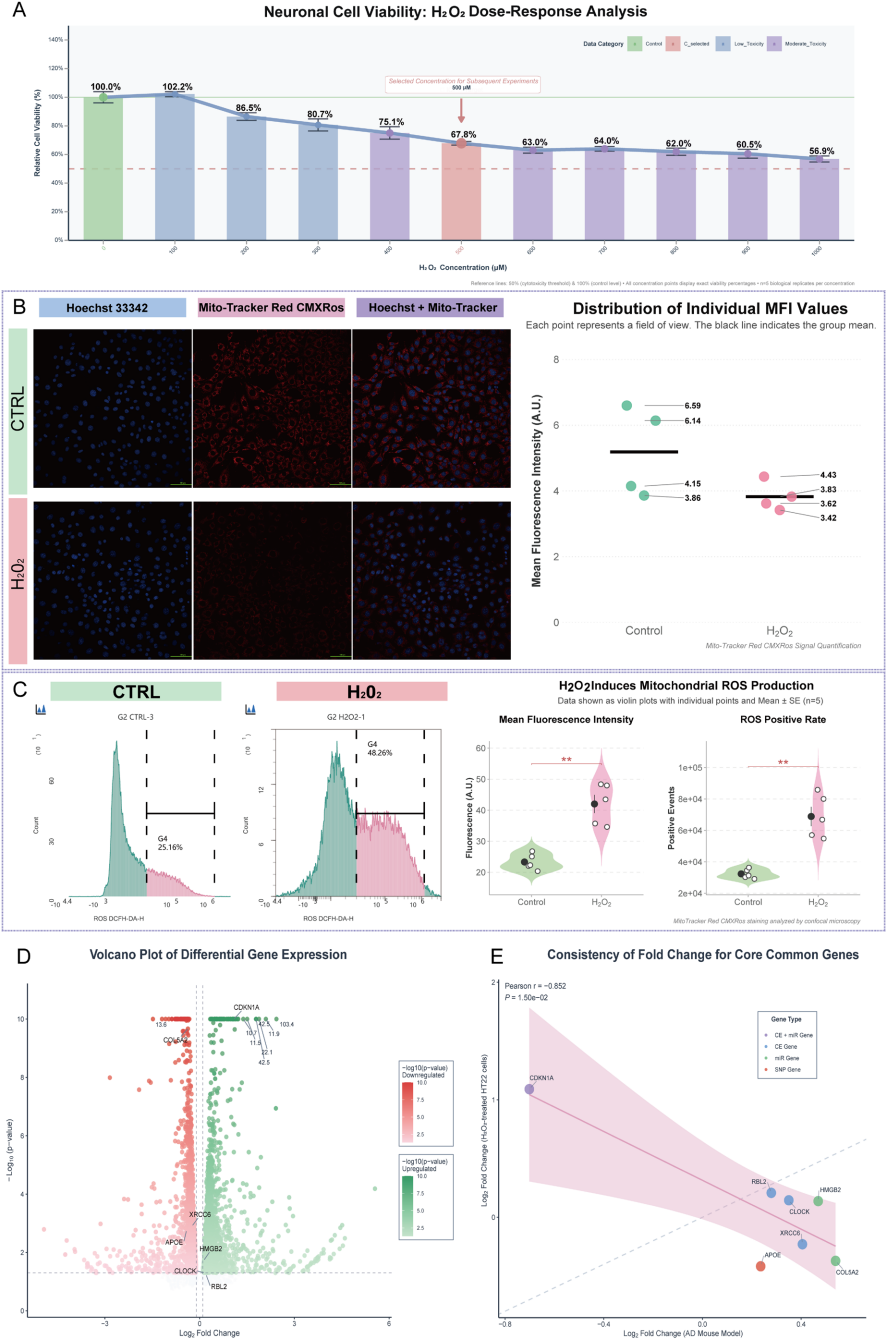
Validation of an H_2_O_2_‐induced oxidative stress model in HT22 cells and its transcriptomic correlation with an AD mouse model. (A) Dose‐dependent suppression of HT22 cell viability by H_2_O_2_ treatment. HT22 cells were exposed to a range of H_2_O_2_ concentrations (0–1000 μM) for 24 h, and cell viability was subsequently quantified using a CCK8 assay. All data are normalized to the untreated control group (0 μM), which is set as 100% viability (solid green line). Results are presented as the mean ± SEM from five independent biological replicates (*n* = 5). A clear dose–response relationship was observed, with viability decreasing as H_2_O_2_ concentration increased. The 50% cytotoxicity threshold, a common benchmark for significant toxicity, is indicated by the dashed red line. Based on these findings, the 500 μM concentration, which induced a substantial and reproducible reduction in cell viability to 67.8% without causing widespread cell death, was selected as the optimal working concentration for subsequent experiments modeling sublethal oxidative stress. This selected concentration is highlighted (red bar and annotation) for clarity. (B) H_2_O_2_ treatment disrupts mitochondrial membrane potential in HT22 cells. HT22 cells, cultured in 20 mm confocal dishes, were treated with 500 μM H_2_O_2_ for 24 h. Following treatment, cells were co‐stained for 30 min with Mito‐Tracker Red CMXRos (1 μM, red, functionally polarized mitochondria) and the nuclear counterstain Hoechst 33342 (1 μM, blue), and subsequently visualized using a Nikon A1 confocal microscope. (Left panels) Representative fluorescence images were captured (Mito‐Tracker Red: Ex/Em ~579/599 nm; Hoechst 33342: Ex/Em ~350/461 nm). A marked reduction in the red fluorescence signal is evident in H_2_O_2_‐treated cells compared to the control group, indicating a loss of mitochondrial membrane potential. Scale bar = 50 μm. (Right panel) Quantitative analysis of the Mean Fluorescence Intensity (MFI) of Mito‐Tracker Red from four independent fields of view (*n* = 4) per group, performed using ImageJ software. Each dot represents the MFI from a single field of view, and the solid black line indicates the group mean. The analysis confirms a significant decrease in mitochondrial fluorescence following H_2_O_2_ exposure. (C) H_2_O_2_ treatment increases intracellular ROS levels in HT22 cells. HT22 cells were treated with or without 500 μM H_2_O_2_ for 24 h. Intracellular reactive oxygen species (ROS) levels were detected using the fluorescent probe DCFH‐DA and analyzed by flow cytometry. (Left) Representative flow cytometry histograms showing the shift in fluorescence intensity. The G4 gate indicates the percentage of ROS‐positive cells. (Right) Violin plots quantifying the mean fluorescence intensity and ROS positive rate (positive events). H_2_O_2_‐induced intracellular ROS identified with fluorescent probe and flow cytometry. Significantly elevated ROS level in H_2_O_2_‐treated cells. Data are shown as individual points with mean ± SE (*n* = 5). **p < 0.01, unpaired *t*‐test. (D) Volcano plot illustrating differentially expressed genes (DEGs) in H_2_O_2_‐treated HT22 cells. The plot shows the distribution of 3516 DEGs identified by RNA‐seq. The x‐axis represents the Log_2_ Fold Change, and the y‐axis represents the statistical significance as –Log_10_(*p* value). Green dots indicate significantly upregulated genes, while red dots indicate significantly downregulated genes. Among the most significantly upregulated genes were CES2 (log_2_FC = 2.43, *p‐ad*j = 5.34 × 10^−100^) and CDKN1A (log_2_FC = 1.09, *p‐ad*j = 3.04 × 10^−72^). Conversely, key downregulated genes included COL3A1 (log_2_FC = −0.88, *p‐adj* = 1.21 × 10^−39^) and H1‐0 (log_2_FC = −0.75, *p‐ad*j = 2.94 × 10^−38^). The labeled genes represent those with the highest statistical significance. (E) Correlation analysis of the Log_2_ Fold Change for seven core common DEGs between the AD mouse model (x‐axis) and H_2_O_2_‐treated HT22 cells (y‐axis). The analysis reveals a significant negative correlation between the two models for the genes APOE, CDKN1A, CLOCK, COL5A2, HMGB2, RBL2, and XRCC6 (Pearson *r* = −0.852, *p* = 0.015).

Given that oxidative stress and mitochondrial dysfunction are core pathological mechanisms in AD, we sought to functionally validate whether the gene signature observed in vivo was responsive to these specific stressors. We first established an in vitro model using HT22 hippocampal neuronal cells. A dose–response analysis demonstrated that H_2_O_2_ induced clear dose‐dependent cytotoxicity, with 500 μM H_2_O_2_ reducing cell viability to a sublethal level of 67.8%. This concentration was selected as it induces significant but recoverable cellular stress, making it ideal for mechanistic studies (Figure 8A, Table S11).

We then directly confirmed that this treatment induced the key pathological features of interest. First, we assessed mitochondrial integrity by measuring the mitochondrial membrane potential (MMP) with Mito‐Tracker Red CMXRos staining. Confocal microscopy revealed that while control cells exhibited a vibrant, intact filamentous mitochondrial network, cells treated with 500 μM H_2_O_2_ for 24 h showed a dramatic, diffuse, and weakened fluorescent signal, indicating widespread collapse of the MMP (Figure 8B, left panels). Quantitative analysis of Mean Fluorescence Intensity (MFI) substantiated these visual findings, showing a significant reduction in the H_2_O_2_‐treated group compared to the control group (mean MFI = 3.82 vs. 5.18 A.U.; Figure 8B, right panel). Second, to confirm the induction of oxidative stress, we measured intracellular reactive oxygen species (ROS) levels using the DCFH‐DA probe and flow cytometry. H_2_O_2_ treatment caused a pronounced rightward shift in the fluorescence histogram, indicative of massive ROS accumulation. Quantitative analysis showed a significant increase in both MFI (23.33 vs. 42.02) and the ROS positive rate (32,365 vs. 68,885 positive events) in treated cells (*p* < 0.01; Figure 8C). Collectively, these experiments rigorously establish that our in vitro model successfully recapitulates the core mechanisms of mitochondrial dysfunction and oxidative stress.

### 3.11 Cross‐Model Transcriptomic Analysis Validates a Conserved Gene Signature Responsive to Oxidative Stress

Having functionally validated our in vitro model, we used it to test our central hypothesis: that the gene signature from our AD mouse model is functionally linked to oxidative stress. RNA‐seq analysis of the H_2_O_2_‐treated HT22 cells identified 3651 DEGs, providing a comprehensive transcriptomic profile of the cellular response to oxidative insult (Figure 8D). We then proceeded with a cross‐model comparative analysis to bridge our in vivo and in vitro findings.

First, within the AD mouse model DEGs, we confirmed significant expression alterations in genes belonging to our computationally prioritized categories. Specifically, nine genes from our predicted ceRNA regulatory network (*ABCG2*, *B4GALT1*, *CDKN1A*, *CLOCK*, *FGF7*, *GJA1*, *KLF6*, *RBL2*, and *XRCC6*), five prominent miRNA target genes (*CDKN1A*, *CFL2*, *GJA1*, *HMGB2*, and *SUZ12*), and three genes linked to PRS‐derived SNPs (*APOE*, *CBLC*, and *ZNF112*) were all found to be significantly dysregulated (Figure 7K). Notably, several of these genes have established mitochondrial associations, such as the mitochondrial membrane‐localized *ABCG2* and *GJA1*, and the mitochondrial‐epistatic genes *CBLC* and *CLOCK* [17, 19], hinting at their potential role in the observed pathology.

The critical validation step involved intersecting the DEGs from the cognitively impaired mouse brain with the DEGs from the oxidatively stressed neuronal cells. This cross‐model analysis revealed a core set of seven genes—*APOE*, *CDKN1A*, *CLOCK*, *COL5A2*, *HMGB2*, *RBL2*, and *XRCC6*—that were consistently and significantly differentially expressed in *both* the chronic in vivo AD model and the acute in vitro oxidative stress model. The conserved dysregulation of this seven‐gene signature across two distinct but mechanistically related models provides powerful functional evidence. It demonstrates that the transcriptomic changes observed in the brains of cognitively impaired mice are directly responsive to the cellular pathways of mitochondrial dysfunction and oxidative stress, thereby validating the relevance of this specific gene set to oxidative stress‐related pathways in AD pathogenesis (Figure 8E, Table S11).

## 4 Discussion

This study focused on the integration of diverse machine learning algorithms to identify mitochondria‐related multi‐omics biomarkers in cognitive decline and neurodegeneration, followed by experimental validation. Acknowledging the complexity of these pathological processes, our approach lies in applying advanced, integrated analytical methods to explore mitochondrial roles in brain aging and cognitive impairment, extending beyond single‐omic or less integrated studies. By leveraging the combined strengths of these algorithms and validating findings through a complementary two‐model system (in vivo D‐galactose/sodium nitrite‐induced aging model and in vitro H_2_O_2_‐induced mitochondrial dysfunction model), we aimed to enhance biomarker precision and explore functional connections between identified signatures and mitochondrial pathways. This integrated computational‐experimental approach provides valuable evidence for understanding mitochondrial contributions to cognitive decline pathogenesis—an important step towards developing more targeted therapeutic interventions addressing mitochondrial dysfunction in neurodegenerative conditions.

Our initial multi‐omics differential expression analyses established a landscape of progressive molecular dysregulation across the AD spectrum. The epigenetic changes were far more extensive in established AD than in MCI, with functional enrichment analyses pointing towards a widespread disruption of neurodevelopmental and synaptic signaling pathways. A critical insight emerged from the transcriptomic data, where the transition from MCI to AD was uniquely marked by a significant enrichment of mitochondrial functions, particularly respiratory chain complex assembly. This finding strongly suggests that a breakdown in mitochondrial bioenergetics is not merely a chronic feature of AD but a key molecular event that accompanies the clinical progression to dementia. This is further supported at a regulatory level, where dysregulated miRNAs in AD were found to preferentially target genes integral to mitochondrial processes, including the chaperone *HSPA1B* and the cell cycle regulator *CDK6*.

Our multi‐omics screening identified key genes, extending their mitochondrial relevance in AD. Notable among these were *HSPA1B* (localized in mitochondria, previously linked to LOAD noncognitive symptoms alongside *HSPA1A* and *HSPA2* [45]; its family member HspB8 inhibits Aβ aggregation [46]). and the mitochondrial epistatic genes *CDK6* (implicated in neuronal cell cycle dysregulation, tau hyperphosphorylation, and neuroinflammation, with the *YAP‐CDK6* axis noted as promising [47]) and *IGF1R* (where defects suggest resistance in AD neurons despite some benefits of IGF‐1 administration [48], supported by correlations of serum IGF‐1 levels with AD risk and resistance [49, 50]). The convergence of multiple dysregulated miRNAs onto this small set of genes implies they are critical, non‐redundant nodes in the AD pathogenic network, suggesting that a coordinated regulatory assault on pathways of mitochondrial function and cell cycle control is a core feature of the disease.

Our survival analysis identified *SLC6A12*, the norepinephrine transporter essential for synaptic signaling [51, 52], as a novel and robust protective biomarker for AD. The significance of this finding is underscored by its consistent, significant protective association with AD risk across both the ROSMAP and ADNI cohorts. While *SLC6A12's* role in cognitive function is established, our novel contribution lies in demonstrating its consistent protective effect across both ROSMAP and ADNI cohorts, positioning it as a robust prognostic biomarker. The strong correlations observed between *SLC6A12* and other genes (*RIPOR3*, *PIK3R1*) in brain tissues suggest the existence of co‐regulatory networks that may collectively influence synaptic resilience and mitochondrial homeostasis in AD. This finding warrants mechanistic studies to elucidate whether *SLC6A12's* protective effect operates through direct mitochondrial mechanisms or indirect synaptic preservation. This finding positions *SLC6A12* not only as a promising prognostic marker but also as a potential therapeutic target. Enhancing its function via noradrenergic modulators could be a viable strategy, with *SLC6A12* expression levels potentially serving as a pharmacodynamic biomarker to monitor treatment response. In parallel with identifying novel protective factors, our integrated pipeline validated the central role of established pathological drivers. Rigorous screening confirmed that *BACE1* expression was a top feature strongly associated with CSF biomarkers of AD pathology. This corroborates its canonical function in Aβ generation [53] and its more recently described role in inducing mitochondrial dysfunction via Klotho cleavage [54]. Our analysis adds a quantitative dimension, revealing strong negative correlations between *BACE1* expression and CSF levels of Aβ, alongside positive correlations with tau and p‐tau. This suggests that peripheral or accessible measurements of *BACE1* activity could serve as a valuable proxy for central pathological load. Thus, beyond reaffirming *BACE1* as a key therapeutic target, our findings provide quantitative biomarker support that could aid in patient stratification and therapeutic monitoring for *BACE1* inhibitor trials.

The machine learning model performance analysis demonstrated that integrating multi‐omics features (3omic model) outperformed single‐omic approaches, with improvements observed in predicting brain resilience indicators based on Aβ and tau profiles. This finding suggests that mitochondrial dysfunction in AD involves coordinated perturbations across multiple molecular layers. The 3omic model achieved AUC values ranging from 0.72 to 0.84 for AD risk prediction, suggesting potential clinical utility for risk assessment when combined with existing diagnostic approaches. Importantly, our analytical framework addresses the fundamental challenge of AD clinical heterogeneity, which has historically limited the translational success of biomarker studies. AD heterogeneity manifests across multiple dimensions, including age of onset (early vs. late), cognitive domains affected (memory‐predominant vs. executive‐predominant), neuropathological burden (amyloid‐positive vs. suspected non‐Alzheimer pathophysiology), and progression rates. Our multi‐omics integration strategy inherently captures this molecular diversity by identifying mitochondrial dysfunction signatures that operate across different pathophysiological axes. The consistent identification of core mitochondrial pathways (Metals and Cofactors, TCA cycle, respiratory electron transport) across both ROSMAP and ADNI cohorts—despite their distinct recruitment strategies, demographic compositions, and clinical assessment protocols—provides compelling evidence that our biomarkers reflect fundamental pathogenic mechanisms rather than cohort‐specific confounders. Furthermore, the superior performance of ensemble machine learning approaches over single algorithms suggests that AD heterogeneity requires computational methods capable of modeling complex, non‐linear interactions between multiple biological systems. Our pathway‐based polygenic risk score approach is particularly innovative in this context, as it acknowledges that genetic susceptibility to mitochondrial dysfunction may manifest through diverse molecular pathways depending on individual genetic architecture, thereby accommodating the polygenic nature of AD risk while maintaining mechanistic coherence.

Co‐expression analysis of GTEx tissues [55] revealed novel, robust brain‐specific correlations (*RIPOR3* vs. *SLC6A12*, *PIK3R1* vs. *CDK6*, and *SLC6A12* vs. *PIK3R1*; Figure S12, Table S12). These findings suggest potential co‐regulatory networks linking *SLC6A12* and *CDK6* (a mitochondrial epistatic gene) to mitochondrial function and cellular homeostasis in AD. The strong correlation of *SLC6A12* with *RIPOR3* and *PIK3R1* points to a previously unrecognized role in AD progression, meriting further investigation into its mechanistic contributions to mitochondrial and synaptic dysfunction.

Our miRNA analysis corroborated emerging research [56, 57], on the downregulation of hsa‐miR‐129‐5p and hsa‐miR‐132 in AD brains. We found that these miRNAs were not only downregulated but also that their higher levels were functionally linked to greater brain resilience, suggesting their loss contributes to a reduced capacity to withstand pathology. The identified miRNA panel (*hsa‐miR‐132*, *hsa‐miR‐129‐5p*, *hsa‐miR‐155‐5p*) demonstrated associations with brain resilience indicators and CSF biomarkers, suggesting potential as minimally invasive diagnostic tools, though further validation is needed. The novelty of our ceRNA network analysis lies in providing a tangible mechanistic framework for these interactions, illustrating regulatory axes such as *hsa‐miR‐132‐3p–NEAT1–hsa_circ_0132052* and *hsa‐miR‐129‐5p–MALAT1–hsa_circ_0066962*. While *NEAT1* and *MALAT1* are known for nuclear paraspeckle regulation and mitochondrial function, respectively [58, 59], our study integrates them into specific AD‐relevant ceRNA networks, suggesting new mitochondrial regulatory pathways. The *MALAT1*/hsa‐miR‐129‐5p interaction, previously noted in other research [60], is here implicated in AD mitochondrial homeostasis. The ceRNA network analysis revealed regulatory nodes (*hsa‐miR‐124‐3p*, *NEAT1*, *PTEN*) that may represent intervention points for mitochondrial‐focused therapeutic development.

MCC scoring further unveiled the significant role of hsa‐miR‐155‐5p, hsa‐miR‐124‐3p, and hsa‐miR‐181a‐5p. While their individual roles in T‐cell function/neuroinflammation (hsa‐miR‐155 [61]), mitochondrial biogenesis (*TFAM* targeting by hsa‐miR‐155 [62]; *INPP5D* association [63]), Aβ reduction/cognitive function (hsa‐miR‐124‐3p via synaptic mitochondria [64, 65]), and memory/glucose metabolism (hsa‐miR‐181a‐5p via mitochondrial ETC [66, 67]) are recognized, our innovation is the comprehensive identification of these miRNAs through MCC scoring within our AD‐specific ceRNA network and, critically, proposing a unifying mitochondrial‐centered perspective for their diverse actions in AD pathogenesis.

The polygenic risk score analysis revealed that the “Metals and Cofactors” pathway‐specific PRS exhibited the most consistent performance across both cohorts, mechanistically aligning with the established role of redox‐active metals (iron, copper, zinc) in mitochondrial dysfunction and oxidative stress in AD [68, 69, 70]. This finding suggests that genetic variants affecting metal homeostasis may represent fundamental vulnerability factors for AD development. The superior performance of the Mitochondrial Central Dogma and Protein Import pathway PRS further emphasizes that disruption of mitochondrial protein synthesis and import machinery represents a core pathogenic mechanism in AD. The MT pathway PRSs, particularly Pt_mt and Pt_inter, showed discriminatory power that could potentially be assessed through standard genotyping platforms, though clinical validation is required.

Cox regression built upon our previous study [17], by confirming Pt_mt, Pt_inter, and additionally identifying Pt_sig as key AD risk contributors, innovatively underscoring the critical regulatory interplay between mitochondrial and nuclear genomes in AD progression across multiple datasets. The correlation patterns between PRSs and clinical phenotypes (MMSE scores, CSF biomarkers) provide a potential framework for precision medicine approaches, pending prospective validation. A robust positive correlation between the brain resilience index and Pt_inter (ROSMAP), and negative correlations of these PRSs with MMSE (ADNI), newly substantiates their capacity to gauge AD severity and resilience from a genetic standpoint.

Significant correlations between MT pathway PRSs (e.g., Pt_mt_res_elec_trans and Pt_CI_assembly_factors) reflect known biological interdependencies like ETC and CI assembly [71, 72] critical for neuronal integrity. Our study innovatively demonstrates that incorporating these mitochondria‐related PRSs, particularly Pt_inter and Pt_TCA_cycle, significantly amplifies the efficacy of machine learning models for brain resilience indicators. This corroborates documented TCA cycle impairments in AD brains [73, 74] and the cycle's homeostatic importance [75], while our novelty lies in showing the predictive power of these specific PRSs and their interactions. Correlation analysis of modeled features unveiled novel insights into functional interplay between PRSs for TCA cycle, phospholipid metabolism, and mtRNA granules, highlighting their collective importance for mitochondrial homeostasis (e.g., TCA precursors for phospholipids, mtRNA granules for TCA enzyme synthesis [76]), whose disruption contributes to AD [7].

Annotation of SNPs within the high‐impact PRSs Pt_inter and Pt_TCA_cycle (an innovation in itself for prioritizing variants) pinpointed lead SNPs: rs714948 (*PVR*, effect on *PVR* expression known [61]), rs57537848 (*NECTIN2*), rs6859 (*NECTIN2*, previously linked to cognitive decline trajectories [77]), and rs620807 (*BLOC1S3*, a significant hit in prior tissue‐specific analyses [78]), with *NECTIN2* levels associated with AD risk [79]. Our study elevates these specific SNPs as key candidates due to their presence in our functionally relevant PRSs (Figure S13A). Examination of mapped genes confirmed the involvement of known players like *APOE*, *APOC1*, *APOC2*, *APOC4* in lipoprotein metabolism and identified numerous zinc finger protein (*ZNF*) family genes (e.g., *ZNF404*) as regulators alongside them. While ZNFs are known for mitochondrial protein import [80], brain development [81, 82], and some links to AD pathology [54], our innovation is the eQTL analysis exposing significant interactions between these SNPs and the mitochondrial epistatic gene *CEACAM19* (itself linked to AD risk via TWAS [83]), suggesting novel co‐regulatory mechanisms involving our identified genetic markers, *CEACAM19*, and established AD genes. Reactome enrichment analysis further innovatively delineated a complex network involving *CEACAM19*, *PVR*, *BCAM*, *APOE*, and these ZNFs in lipoprotein regulation and *NR1H3/NR1H2* signaling. This suggests a previously uncharacterized shared regulatory influence of mitochondria, ZNFs, and *APOE* on AD pathogenesis, illuminated by our integrated approach (Figure S13B).

Our experimental validation strategy provided multiple layers of evidence for the clinical relevance of computationally identified biomarkers. The D‐galactose–induced AD mouse model demonstrated significant cognitive impairment and brain remodeling, establishing a valid experimental framework for testing our predictions. The RNA‐seq analysis of AD mouse brain tissue validated the differential expression of multiple genes from our ceRNA network (*ABCG2*, *B4GALT1*, *CDKN1A*, *CLOCK*, *FGF7*, *GJA1*, *KLF6*, *RBL2*, *XRCC6*), miRNA targets (*CDKN1A*, *CFL2*, *GJA1*, *HMGB2*, *SUZ12*), and PRS‐derived genes (*APOE*, *CBLC*, *ZNF112*), confirming the pathogenic relevance of our multi‐omics findings. The complementary H_2_O_2_‐treated HT22 cell model provided crucial insights into the oxidative stress responsiveness of our identified genes. The overlap of seven genes (*APOE*, *CDKN1A*, *CLOCK*, *COL5A2*, *HMGB2*, *RBL2*, *XRCC6*) between chronic neurodegenerative conditions and acute oxidative stress demonstrates the conserved transcriptional response patterns and validates their relevance to oxidative stress‐related pathways in AD pathogenesis. This cross‐model validation approach strengthens the translational potential of these biomarkers by showing their responsiveness across different experimental contexts and time scales. Among the validated genes, several demonstrated established mitochondrial associations, including mitochondrial membrane‐localized *ABCG2* and *GJA1*, and mitochondrial‐epistatic genes *CBLC* and *CLOCK*. This experimental confirmation supports our computational predictions and provides evidence that these genes represent robust candidates for monitoring therapeutic interventions targeting mitochondrial dysfunction and oxidative stress in AD. The dysregulation of several key genes validated in our study points towards a multifactorial mechanistic landscape in AD pathogenesis. For instance, *CDKN1A* (p21), a potent cyclin‐dependent kinase inhibitor, reflects a cellular response to the persistent genotoxic and oxidative stress prevalent in the AD brain. Beyond its canonical role in enforcing cell cycle arrest, *CDKN1A* is now understood to be a direct modulator of mitochondrial integrity and cell fate [84]. Elevated p21 levels can promote mitochondrial dysfunction and sensitize cells to apoptosis, partly by altering the transcriptional balance of Bcl‐2 family proteins which govern mitochondrial outer membrane permeabilization (MOMP) [85]. This p21‐driven senescent state also fuels the neuroinflammatory environment through the senescence‐associated secretory phenotype (SASP), a process shown to be dependent on mitochondrial function and ROS signaling, thereby creating a destructive feedback loop [86].

The altered expression of *CLOCK*, a core component of the circadian pacemaker, provides a direct link between the systemic disruption of biological rhythms and mitochondrial homeostasis at the cellular level. A robust body of evidence demonstrates that the molecular clockwork, orchestrated by *CLOCK* and its partner BMAL1, imposes a critical temporal regulation on mitochondrial bioenergetics [87]. *CLOCK* directly controls the rhythmic expression of numerous nuclear‐encoded genes essential for oxidative phosphorylation, mitochondrial dynamics (fission/fusion), and antioxidant defense pathways [88]. Consequently, its dysregulation in our models likely signifies a breakdown in the daily coordination of mitochondrial energy production and quality control, leading to a state of chronic oxidative stress, impaired metabolic flexibility, and ultimately, neuronal vulnerability [89, 90].

Our analysis also revealed the differential expression of *COL5A2*, a gene encoding a subunit of type V collagen. While collagens are traditionally associated with the peripheral extracellular matrix (ECM), their expression and functional roles within the central nervous system are increasingly recognized [91]. Type V collagen is a crucial regulator of the assembly and diameter of other major collagen fibrils, thereby influencing the structural integrity of the ECM. In the brain, the ECM provides not only structural support but also critical signaling cues that regulate synaptic plasticity, axon guidance, and the inflammatory response [92]. Recent studies have demonstrated that the brain's ECM is significantly remodeled in AD, contributing to synaptic loss and the sequestration of amyloid plaques [93, 94]. Although a direct link between *COL5A2* and mitochondria is not yet established, ECM‐integrin signaling is known to modulate intracellular pathways that impact mitochondrial function and cell survival, such as the PI3K/Akt pathway [95]. Therefore, the altered expression of *COL5A2* may reflect a pathological remodeling of the neuronal microenvironment that indirectly compromises mitochondrial health and synaptic stability.

While this study provides significant insights into the mitochondrial landscape of AD through an innovative integration of multi‐omics data and machine learning, several limitations should be acknowledged. Firstly, the primary reliance on datasets from European‐ancestry populations may limit the generalizability of our findings to other ethnic groups; future studies should encompass more diverse cohorts to validate these mitochondrial signatures globally. While our cross‐cohort validation addresses some aspects of AD clinical heterogeneity, research cohorts may not fully capture community‐based populations with diverse socioeconomic backgrounds and comorbidity profiles. Secondly, our analysis focuses on elderly populations, which limits our understanding of early‐life or mid‐life mitochondrial changes that may contribute to AD pathogenesis. While this age focus was necessary given the availability of well‐characterized AD cohorts and the need to study established disease states, it restricts our ability to identify early biomarkers or optimal intervention windows that may occur decades before symptom onset. Thirdly, although advanced machine learning algorithms and rigorous cross‐validation techniques were employed, the inherent complexity and sample sizes typical of multi‐omics integration studies mean that the possibility of model overfitting or the identification of spurious associations cannot be entirely excluded, necessitating independent replication. The optimal integration of multi‐omics data for capturing the full spectrum of AD pathophysiology remains an evolving challenge. Thirdly, the cross‐sectional nature of some analyses, while valuable for biomarker discovery, does not fully capture the dynamic temporal changes of these mitochondrial features throughout AD progression; longitudinal studies are required to delineate the trajectory of these alterations. This is particularly relevant given AD's heterogeneous progression patterns, where individuals may follow distinct trajectories requiring personalized approaches. Finally, while our in vivo and in vitro validation strengthens the translational relevance of identified genes, further mechanistic studies are essential to fully elucidate the functional roles of these novel mitochondrial‐associated players and networks in AD pathogenesis. Translation from mouse models to human AD involves inherent complexity due to species differences and the multifactorial nature of human disease. Similarly, our in vitro experiments, while functionally linking identified genes to mitochondrial health under oxidative stress, relied on a single immortalized murine cell line (HT22) and an acute H_2_O_2_‐induced stress model. This simplified system does not fully recapitulate the chronic, multifaceted pathology of human AD, which involves complex interactions between various brain cell types (e.g., neurons, glia) and a wider array of pathological insults beyond acute oxidative stress. Addressing these limitations will be crucial for translating our findings into robust clinical applications.

In conclusion, through integrating multi‐omics data with machine learning and validating findings across multiple experimental models, this study has identified and experimentally confirmed mitochondria‐related signatures in AD. Our computational predictions were validated through in vivo AD mouse models and in vitro oxidative stress systems, demonstrating the robustness and translational potential of the identified biomarkers. We characterized novel gene associations with AD (particularly *CDKN1A* and *CLOCK* in mitochondrial contexts), delineated AD‐relevant ceRNA networks, and demonstrated the utility of mitochondria‐specific polygenic risk scores. The convergent evidence from human cohort data, mouse models, and cellular systems provides a comprehensive foundation for understanding mitochondrial dysfunction in AD pathogenesis and developing targeted therapeutic interventions. These findings offer a validated framework for future diagnostic applications and drug development, with specific biomarkers ready for clinical translation studies.

## Author Contributions

X.X. performed the main analytical and visual tasks. X.X., Y.S., J.L., H.W., and Y.H. contributed to omics data collection and processing. X.‐Y.M. and S.‐S.F. contributed to animal experiments validation. X.X., X.‐Y.M., Y.S., S.‐S.F., J.L., H.W., Y.H., and J.H. contributed to drafting the manuscript. X.X., X.‐Y.M., and Y.S. led the preparations of the manuscript and provided the overall supervision of the project. All authors reviewed and approved the accepted version of the manuscript.

## Disclosure

Code Availability: Essential scripts for implementing machine learning‐based integrative procedures and visualize are available on the GitHub website (https://github.com/XuanXu1230/ML‐AD).

## Ethics Statement

All procedures conducted in this study, which involved human participants, adhered to the ethical standards set forth by the institutional and national research committee. Furthermore, the research was performed in compliance with the 1964 Helsinki Declaration and its subsequent amendments, or equivalent ethical guidelines. This study relied on publicly available data from published research, necessitating no individual‐level data collection. As such, no new ethical review was required, as the original studies had ethical clearance and participant consent. Data used in this article were obtained from the Alzheimer's Disease Neuroimaging Initiative (ADNI) database, the Religious Orders Study (ROS), and the Rush Memory and Aging Project (MAP) project. The results published here are in whole or in part based on data obtained from the AD Knowledge Portal (https://adknowledgeportal.org). Our research adheres to the data use agreements. For comprehensive details on the ethical considerations and access procedures for these datasets, the ADNI website (https://adni.loni.usc.edu/) and the ROSMAP website (https://www.radc.rush.edu/) serve as authoritative resources. All the animal experiments were ethically approved by the Institutional Review Board of Hubei Minzu University.

## Consent

The authors have nothing to report.

## Conflicts of Interest

The authors declare no conflicts of interest.

## Supporting information


**Figures S1–S13:** cns70634‐sup‐0001‐FigureS1‐S13.docx.

**Tables S1–S12:** cns70634‐sup‐0002‐TableS1‐S12.xlsx.

## Data Availability

The genetic data and gene expression data for co‐expression network construction can be applied from the ADNI website (http://adni.loni.usc.edu). The genetic data, gene and miRNA expression profiles, and post‐mortem pathological measurements of AD patients from ROSMAP can be obtained through RADC (https://www.radc.rush.edu) or the Acknowledge Portal (www.synapse.org). The FUMA database can be accessed through https://fuma.ctglab.nl/. The starBase database can be accessed through https://rnasysu.com/encori/. The circRNA interactions can be obtained from the CircBank database (http://www.circbank.cn/).
