# Supplementary material for: Integrated Multi‐Omics Analysis and Cross‐Model Validation Reveal Mitochondrial Signatures in Alzheimer's Disease
Source: CNS Neurosci Ther. 2025 Oct 27;31(10):e70634. doi: 10.1111/cns.70634 (PMC12559030; doi:10.1111/cns.70634)
Supplement: Supplementary file 1 — Figures S1–S13: cns70634‐sup‐0001‐FigureS1‐S13.docx. [file CNS-31-e70634-s002.docx]

## Description of Supplementary Figures

[**Supplementary Fig.1. Differential methylation analysis reveals distinct epigenetic signatures across AD spectrum in ROSMAP cohort.** 2](#_Toc209356289)

[**Supplementary Fig.2. Differential gene expression analysis reveals distinct transcriptional signatures and mitochondrial dysfunction in AD progression.** 5](#_Toc209356290)

[**Supplementary Fig.3. Differential miRNA expression analysis reveals distinct miRNA signatures and mitochondrial dysfunction in AD progression.** 8](#_Toc209356291)

[**Supplementary Fig.4 Functional annotation cluster analysis reveals distinct biological processes associated with differentially methylated positions across the AD spectrum.** 11](#_Toc209356292)

[**Supplementary Fig.5 Gene ranking and GSEA reveal distinct patterns of gene regulation and pathway activation across the AD spectrum.** 13](#_Toc209356293)

[**Supplementary Fig.6 Integrative approach for identifying and validating important predictive features across multiple models and predictors.** 15](#_Toc209356294)

[**Supplementary Fig.7 Survival and hazard ratio analysis of candidate biomarkers in the ROSMAP dataset reveals significant associations with AD risk across multiple omics layers.** 17](#_Toc209356295)

[**Supplementary Fig.8 Validation of key biomarker associations in ADNI confirms and expands findings from ROSMAP.** 19](#_Toc209356296)

[**Supplementary Fig.9 Correlation analysis in the ROSMAP reveals strong associations between candidate multi-omics features and their potential role in brain resilience.** 21](#_Toc209356297)

[**Supplementary Fig.10 Correlation analysis in the ADNI dataset reveals potential features interactions and associations with AD pathology.** 23](#_Toc209356298)

[**Supplementary Fig.11 Comprehensive annotation of mitochondrial-related SNPs unveil their potential regulatory roles in AD pathogenesis.** 25](#_Toc209356299)

[**Supplementary Fig.12 Tissue-specific co-expression analysis of candidate interacting genes reveals pronounced correlations in brain tissue.** 28](#_Toc209356300)

[**Supplementary Fig.13 Mitochondrial-related biomarker landscape illustrates complex interactions and regulatory networks in AD pathogenesis.** 30](#_Toc209356301)


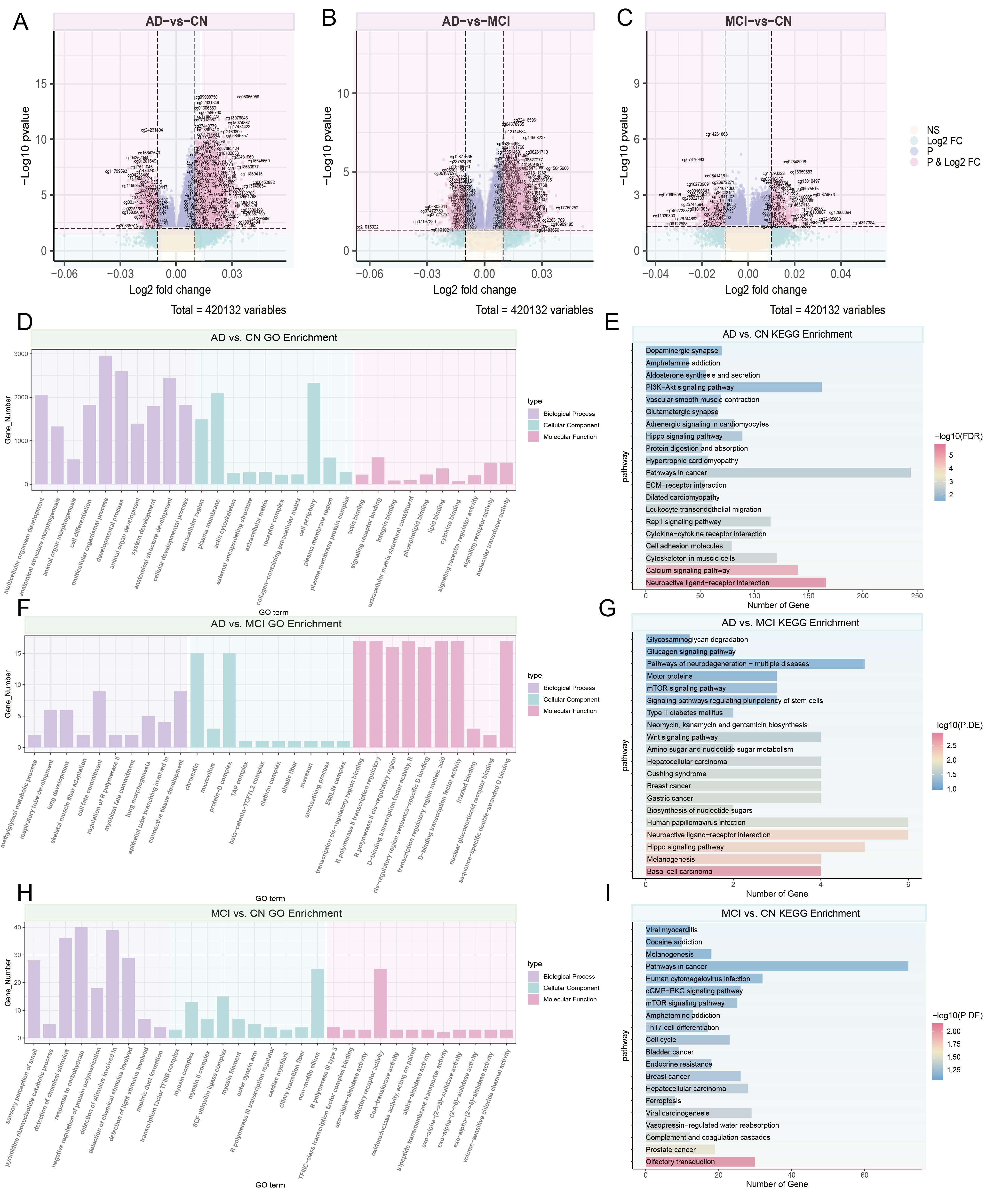


## Supplementary Fig.1. Differential methylation analysis reveals distinct epigenetic signatures across AD spectrum in ROSMAP cohort.

(A-C) Volcano plots illustrating differentially methylated positions (DMPs) in pairwise comparisons. The x-axis represents the log2 fold change in methylation, and the y-axis shows the -log10 P-value for statistical significance. (A) The comparison between AD and cognitively normal (CN) individuals revealed 19,713 significant DMPs (adj.P < 0.01), indicating extensive epigenetic dysregulation in AD. (B) The AD vs. mild cognitive impairment (MCI) comparison identified a more focused set of 128 DMPs (adj.P < 0.01), highlighting key changes during the progression to AD. Notably, 117 of these were shared with the AD vs. CN group. (C) The MCI vs. CN comparison, representing early-stage changes, identified 2,699 DMPs (P < 0.01) , highlighting that substantial epigenetic alterations are already present in the early, preclinical stages of cognitive decline. (D-E) Functional enrichment analysis of DMPs from the AD vs. CN comparison.
(D) This GO enrichment bar plot, categorized by Biological Process (purple), Cellular Component (green), and Molecular Function (pink), shows that DMPs in the AD stage are significantly enriched in fundamental developmental processes. For example, animal organ morphogenesis (GO:0009653, adj.P = 1.33×10⁻²⁸) and organ development (GO:0007275, adj.P = 1.45×10⁻²⁴) were among the most significant terms, suggesting a dysregulation of pathways involved in maintaining tissue structure and cellular identity. (E) The corresponding KEGG pathway analysis reveals enrichment in pathways essential for neuronal function. Specifically, neuroactive ligand-receptor interactions (hsa04080, adj.P = 1.41×10⁻⁶) and calcium signaling pathways (hsa04020, adj.P = 8.42×10⁻⁶) were highly enriched, underscoring that the advanced stage of AD is characterized by widespread disruption of synaptic communication and intracellular signaling. (F-G) Functional enrichment analysis of DMPs from the AD vs. MCI comparison. (F) The GO enrichment analysis for this transitional stage highlights terms related to transcriptional regulation, such as methyl-CpG binding and regulation of transcription from RNA polymerase II promoter. This indicates that the progression from MCI to AD is marked by critical epigenetic modifications that directly influence gene expression control. (G) The KEGG analysis further supports this, showing enrichment in Pathways of neurodegeneration and the Wnt signaling pathway. This suggests that as cognitive decline accelerates, the affected epigenetic loci are increasingly concentrated in pathways central to neuronal survival and developmental signaling. (H-I) Functional enrichment analysis of DMPs from the MCI vs. CN comparison. (H) The GO enrichment for these early-stage DMPs points to alterations in sensory and cellular response pathways. Terms such as sensory perception of smell, detection of chemical stimulus involved in sensory perception, and cilium movement were enriched, consistent with clinical findings of olfactory dysfunction as an early AD symptom. (I) Similarly, the KEGG pathway analysis highlights Olfactory transduction. The enrichment of pathways like the cGMP-PKG signaling pathway and Cocaine addiction may reflect early dysregulation in reward and signaling systems within the brain. These findings collectively suggest that the initial epigenetic landscape changes in AD may target specific sensory and signaling networks before the onset of widespread neurodegeneration.


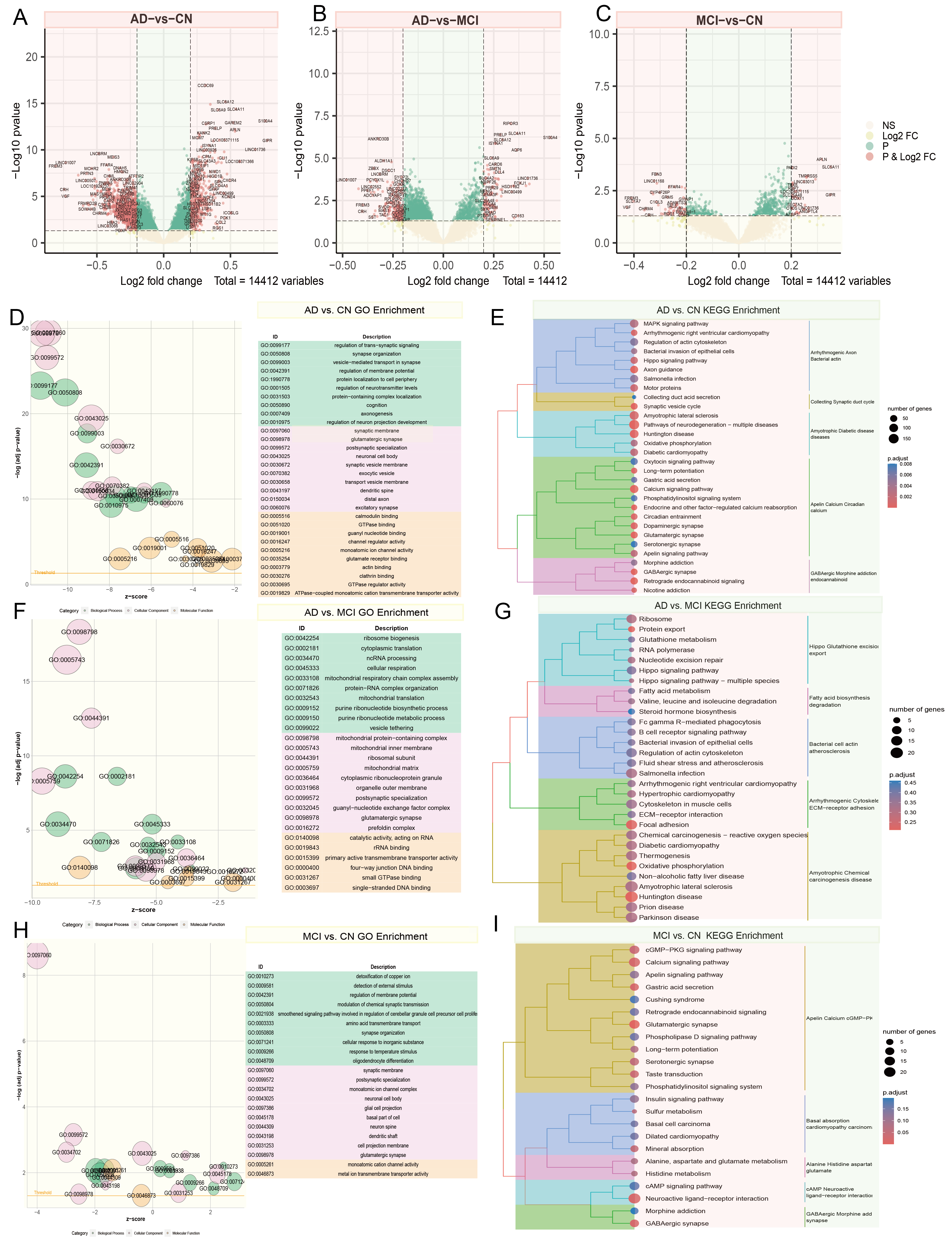


## Supplementary Fig.2. Differential gene expression analysis reveals distinct transcriptional signatures and mitochondrial dysfunction in AD progression.

(A-C) Volcano plots displaying differentially expressed genes (DEGs) in the mRNA dataset. (A) The AD vs. CN comparison, utilizing an adjusted P-value threshold of 0.05, identified a substantial 4,710 DEGs, indicative of widespread transcriptional alterations in the AD brain. (B) In contrast, the AD vs. MCI comparison revealed a more focused set of 49 DEGs, with 48 of these being shared with the AD vs. CN group, suggesting their critical role in the transition from MCI to AD. (C) The MCI vs. CN comparison identified 128 DEGs, highlighting the initial transcriptional changes that occur with the onset of cognitive impairment. Three genes, CCDC69, SLC6A12, and SLC6A9, were differentially expressed across all three comparison groups, marking them as persistent markers of disease progression. (D-E) Functional enrichment of DEGs from the AD vs. CN comparison. (D) This GO enrichment bubble plot shows that DEGs are significantly enriched in biological processes fundamental to neuronal communication. Key enriched terms include regulation of trans-synaptic signaling, regulation of membrane potential, and cellular components like glutamatergic synapse, underscoring a profound disruption of synaptic integrity in AD. (E) The corresponding KEGG pathway analysis, displayed as a clustered dot plot, confirms these findings, showing strong enrichment in glutamatergic synapse, GABAergic synapse, and calcium signaling pathways. Additionally, enrichment in the circadian rhythm and apelin signaling pathways highlights the disruption of broader regulatory networks. (F-G) Functional enrichment of DEGs from the AD vs. MCI comparison highlights mitochondrial dysfunction. (F) The GO enrichment analysis for this transitional stage reveals a striking enrichment of mitochondria-related functions. Specific and highly significant terms include mitochondrial respiratory chain complex assembly (GO:0033108, adj.P = 0.00075) and localization to the mitochondrial inner membrane (GO:0005743, P = 0.00015). This strongly indicates that mitochondrial dysfunction is a key molecular event during the progression from MCI to AD. (G) The KEGG analysis further shows enrichment in pathways such as Ribosome, Fatty acid metabolism, and Pathways of neurodegeneration, reflecting a state of cellular stress involving protein synthesis, energy metabolism, and cell survival pathways as the disease advances.. (H-I) Functional enrichment of DEGs from the MCI vs. CN comparison points to early synaptic and glial alterations. (H) The GO enrichment of the 128 DEGs in this early stage shows involvement in processes crucial for neural integrity, such as regulation of membrane potential, cellular response to extracellular stimulus, and glial-related processes like oligodendrocyte differentiation. (I) The KEGG analysis identifies enrichment in critical signaling pathways, including the apelin signaling pathway, cGMP-PKG signaling pathway, and neuroactive ligand-receptor interaction. These findings suggest that subtle but significant dysregulation of synaptic signaling and intercellular communication occurs at the earliest stages of cognitive decline.


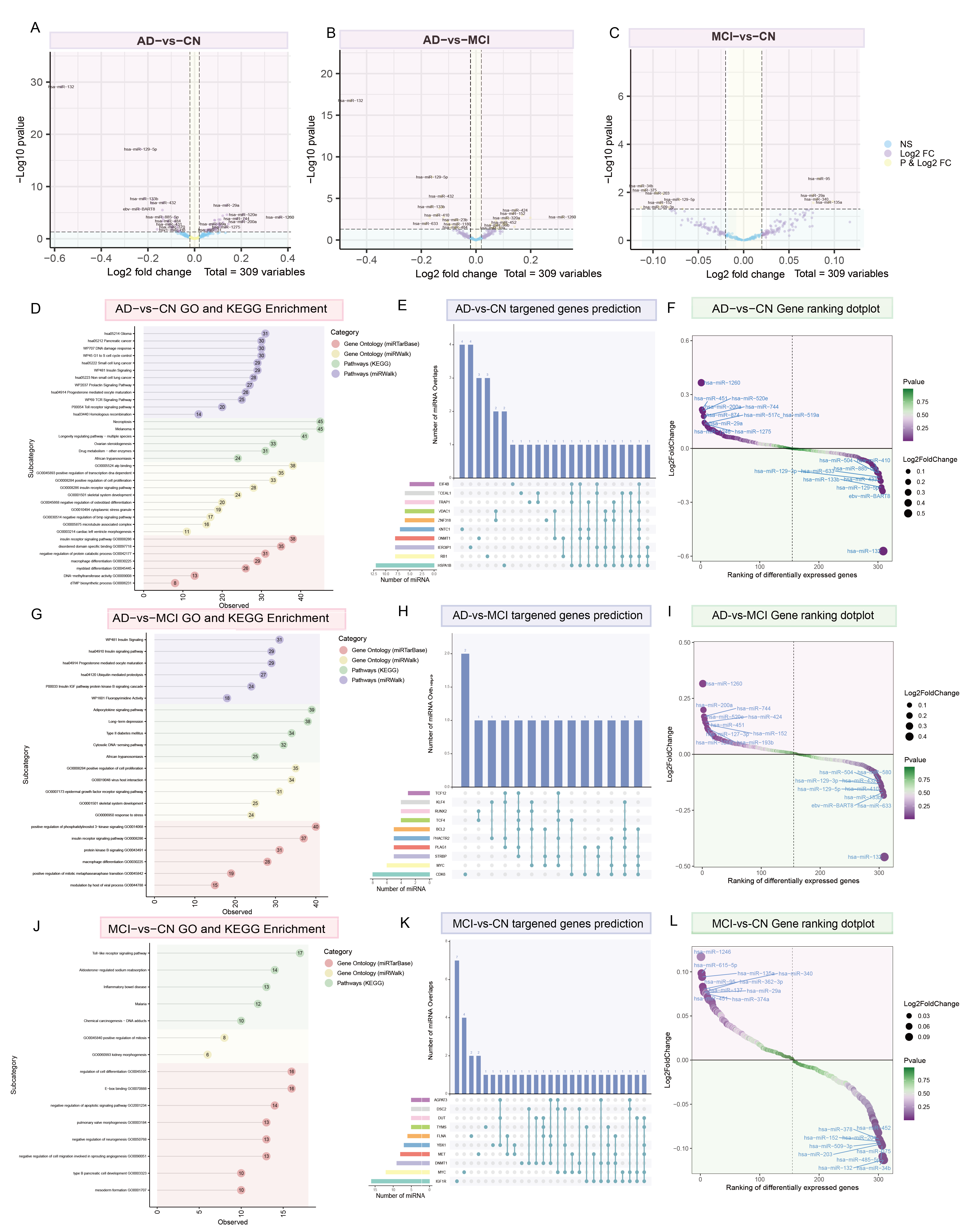


## Supplementary Fig.3. Differential miRNA expression analysis reveals distinct miRNA signatures and mitochondrial dysfunction in AD progression.

(A-C) Volcano plots showing differentially expressed miRNAs (DEMs) identified using an adjusted P-value threshold of 0.01. (A) The AD vs. CN comparison revealed 29 DEMs, indicating a significant alteration of the miRNA regulatory landscape in AD. (B) A smaller set of six DEMs was identified in the AD vs. MCI comparison. Intriguingly, these six DEMs were a subset of those found in the AD vs. CN group, suggesting their involvement in the progression from MCI to AD. (C) The MCI vs. CN comparison identified three DEMs, with two (hsa-miR-95 and hsa-miR-375) also being significant in the AD vs. CN group, highlighting their potential as early-stage biomarkers.

(D-F) Functional characterization of DEMs in the AD vs. CN comparison. (D) The GO and KEGG enrichment plot shows that predicted target genes are significantly enriched in biologically relevant pathways, notably the insulin receptor signaling pathway (GO:0008286, adj.P = 2.14×10⁻⁸) and DNA-methyltransferase activity (GO:0009008, adj.P = 3.08×10⁻⁸), highlighting miRNA-mediated dysregulation of metabolic signaling and epigenetic control in AD. (E) The UpSet plot illustrates that key genes are targeted by multiple dysregulated miRNAs. Genes targeted by the highest number of DEMs include HSPA1B (a mitochondrial localization gene), CDK6, and IGF1R (both mitochondrial epistatic genes), strongly suggesting a coordinated miRNA-led assault on mitochondrial function. (F) The expression ranking dot plot visualizes the log2 fold change of all miRNAs, showing that miRNAs like hsa-miR-1260 are among the most significantly upregulated, while hsa-miR-132 is among the most downregulated, indicating a profound shift in post-transcriptional regulators. (G-I) Functional characterization of DEMs in the AD vs. MCI comparison. (G) Pathway enrichment for this transitional stage shows that DEM targets are involved in insulin signaling and phosphatidylinositol signaling regulation, indicating that dysregulation of these metabolic and signaling pathways is a key feature of disease progression. (H) The UpSet plot confirms that mitochondrial-related genes like HSPA1B and CDK6 remain central targets, reinforcing the hypothesis that mitochondrial pathways are continuously impacted as the disease worsens. (I) The ranking plot shows that the same key miRNAs, such as hsa-miR-1260 (upregulated) and hsa-miR-132 (downregulated), are also prominent in this comparison, solidifying their role as consistent markers of disease progression. (J-L) Functional characterization of DEMs in the MCI vs. CN comparison. (J) Enrichment analysis for this early stage reveals that DEM targets are involved in the negative regulation of cell differentiation and neurogenesis, suggesting that the earliest miRNA alterations may disrupt fundamental processes of cellular maintenance. (K) The UpSet plot for the three DEMs in this group shows their predicted targets, beginning to form the regulatory patterns that become more complex in later disease stages. (L) The ranking plot highlights the subtle but significant expression changes of the three DEMs, representing the initial shift in the miRNA landscape that precedes overt cognitive decline.


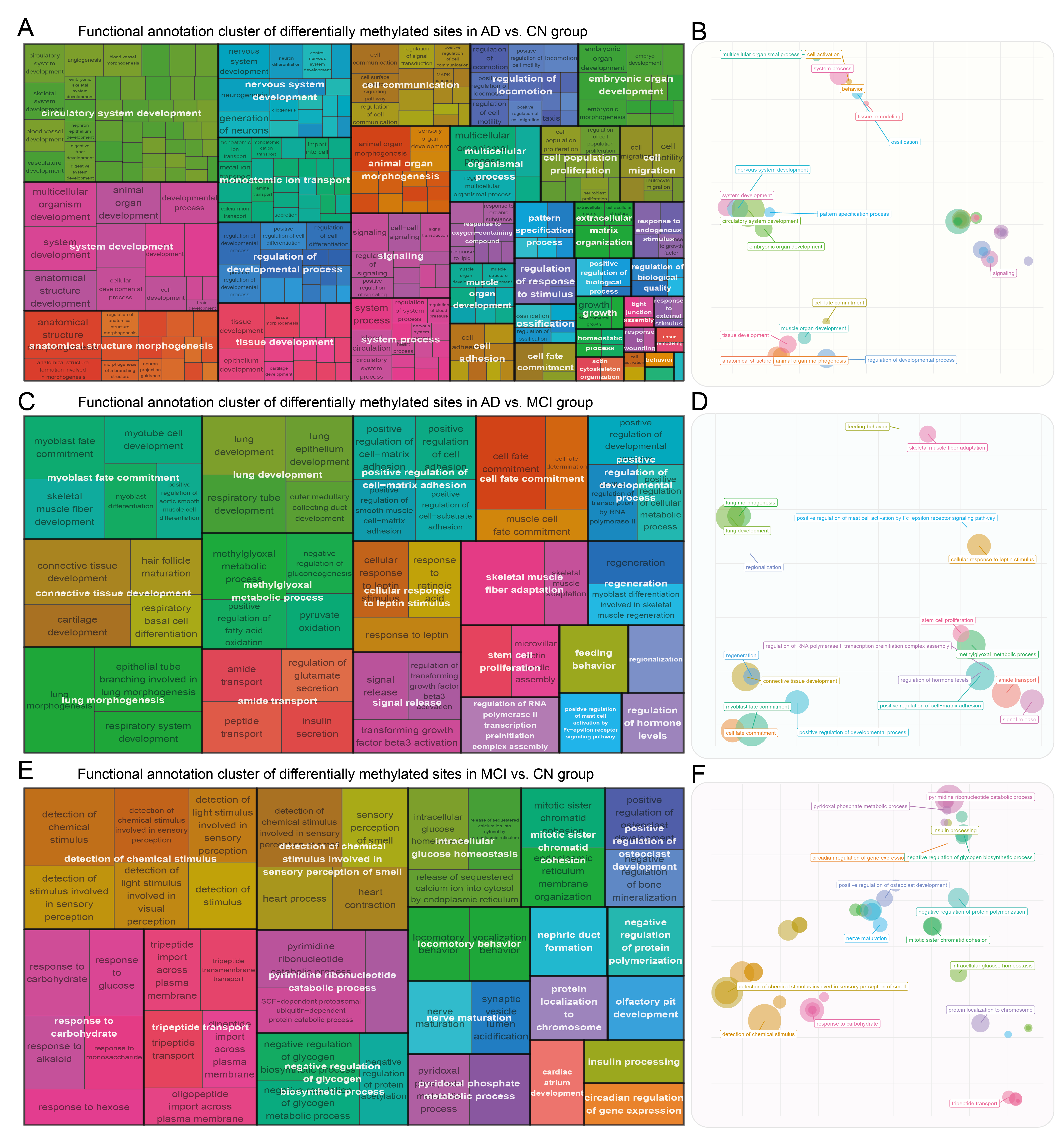


## Supplementary Fig.4 Functional annotation cluster analysis reveals distinct biological processes associated with differentially methylated positions across the AD spectrum.

The dendrogram and cluster analyses elucidate the intricate associations between DMPs and enriched biological processes across the three comparison groups. (A-B) In the AD vs. CN group, DMPs were predominantly linked to the regulation of developmental processes, encompassing the maturation and organization of the circulatory and nervous systems, as well as tissue morphogenesis. These processes are of critical importance for maintaining the structural and functional integrity of tissues essential for cognitive function The enrichment of these processes suggests that the pathological changes observed in AD may disrupt the epigenetic regulation of key developmental pathways, potentially contributing to the cognitive decline and neurodegeneration observed in the disease. (C-D) The AD vs. MCI group exhibited DMPs primarily associated with the regulation of cell fate, connective tissue development, and glyoxal metabolism. The prominence of cell fate regulation suggests that epigenetic alterations may influence cellular decisions between survival, growth, and death during the progression from MCI to AD This finding highlights the potential role of aberrant epigenetic regulation in the neurodegenerative cascade. The involvement of connective tissue development and glyoxal metabolism may reflect the systemic impact of AD and the potential contribution of metabolic dysregulation to the disease pathogenesis. (E-F) The MCI vs. CN group displayed enrichment in processes related to the organism's hardening response to chemical stimuli and carbohydrate reactions. These processes may represent an early adaptive or protective mechanism in response to incipient cognitive decline. The epigenetic regulation of these processes indicates that alterations in cellular signaling and metabolic pathways may occur during the early stages of cognitive impairment, potentially serving as a compensatory response to the underlying pathological changes.


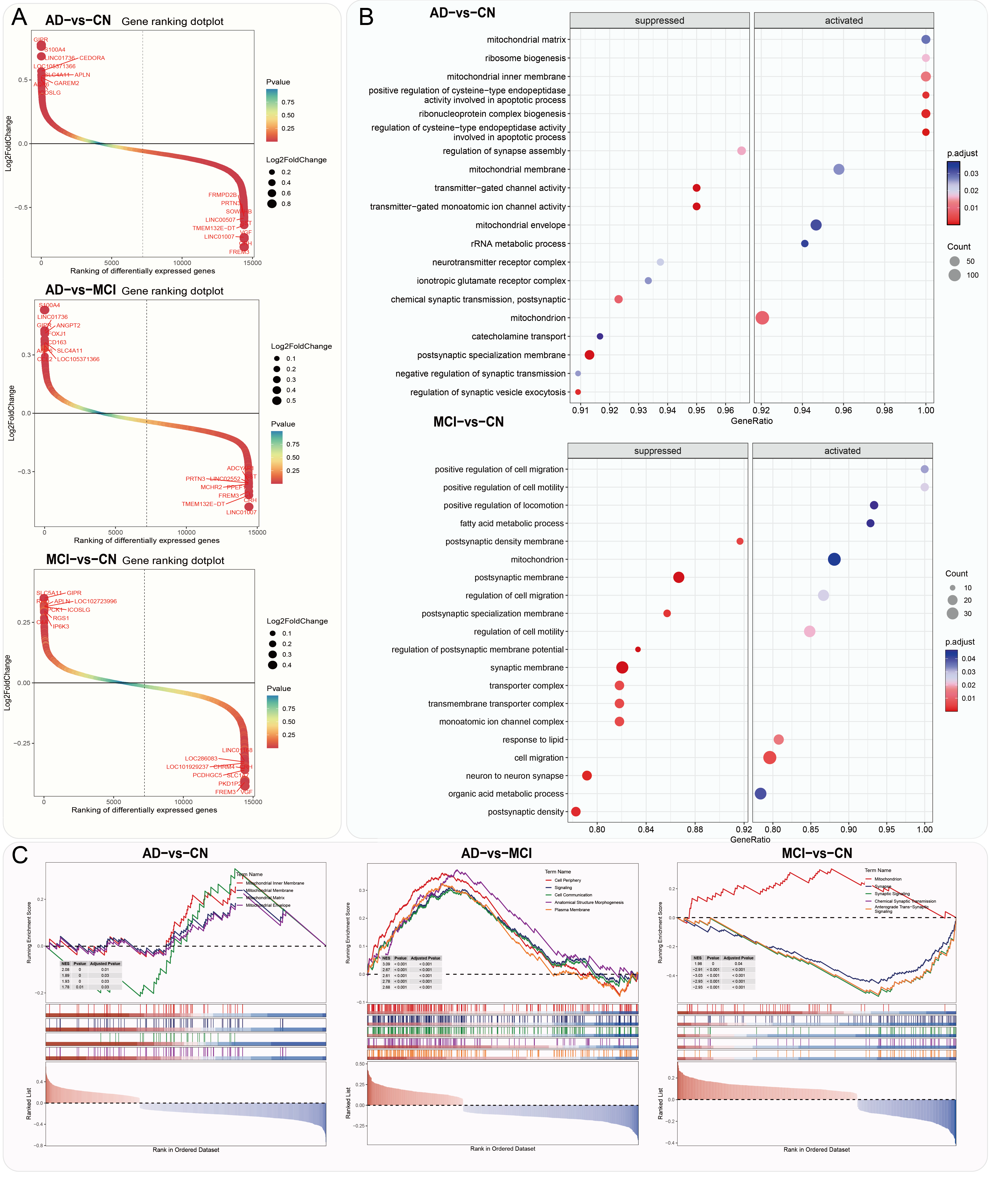


## Supplementary Fig.5 Gene ranking and GSEA reveal distinct patterns of gene regulation and pathway activation across the AD spectrum.

(A) Gene ranking analysis revealed a significant disparity in gene regulation between the AD vs. CN group, with notable upregulation and downregulation events. The most substantially upregulated genes included *GIRP, S100A4*, and *LINC01736*, while the most significantly downregulated included *LINC01007, CRH,* and *TMEM123E*. Notably, a subset of the genes with the highest differential expression ranks in the AD vs. CN comparison, such as *S100A4* (upregulated), and *LINC01007* (downregulated), also exhibited similar trends in the AD vs. MCI comparison. This suggests that these genes are consistently involved in the disease progression spectrum. (B) GSEA revealed distinct patterns of activation and inhibition patterns of genes associated with mitochondrial localization in the AD vs. CN and MCI vs. CN groups. The most pronounced activation was observed in genes related to caspase-like endopeptidase activity and ribosomal protein biogenesis, during the apoptotic process. This indicates their potential role in regulating cell death mechanisms in AD. In the MCI vs. CN group, the most significant inhibition was observed in genes localized to the synaptic membrane and involved in transport protein complexes, suggesting a disruption in synaptic function and vesicular transport, which are critical for maintaining neuronal communication. Concurrently, a notable activation of mitochondrial genes was observed, which may reflect a compensatory response to the metabolic and energetic demands of the neurons in the early stages of cognitive decline. (C) A closer examination of the mitochondrial pathways revealed heightened activation in the AD vs. CN group, while the AD vs. MCI group exhibited activation of pathways related to cell signaling and communication. This may represent an adaptive cellular response to the initial cognitive impairments.


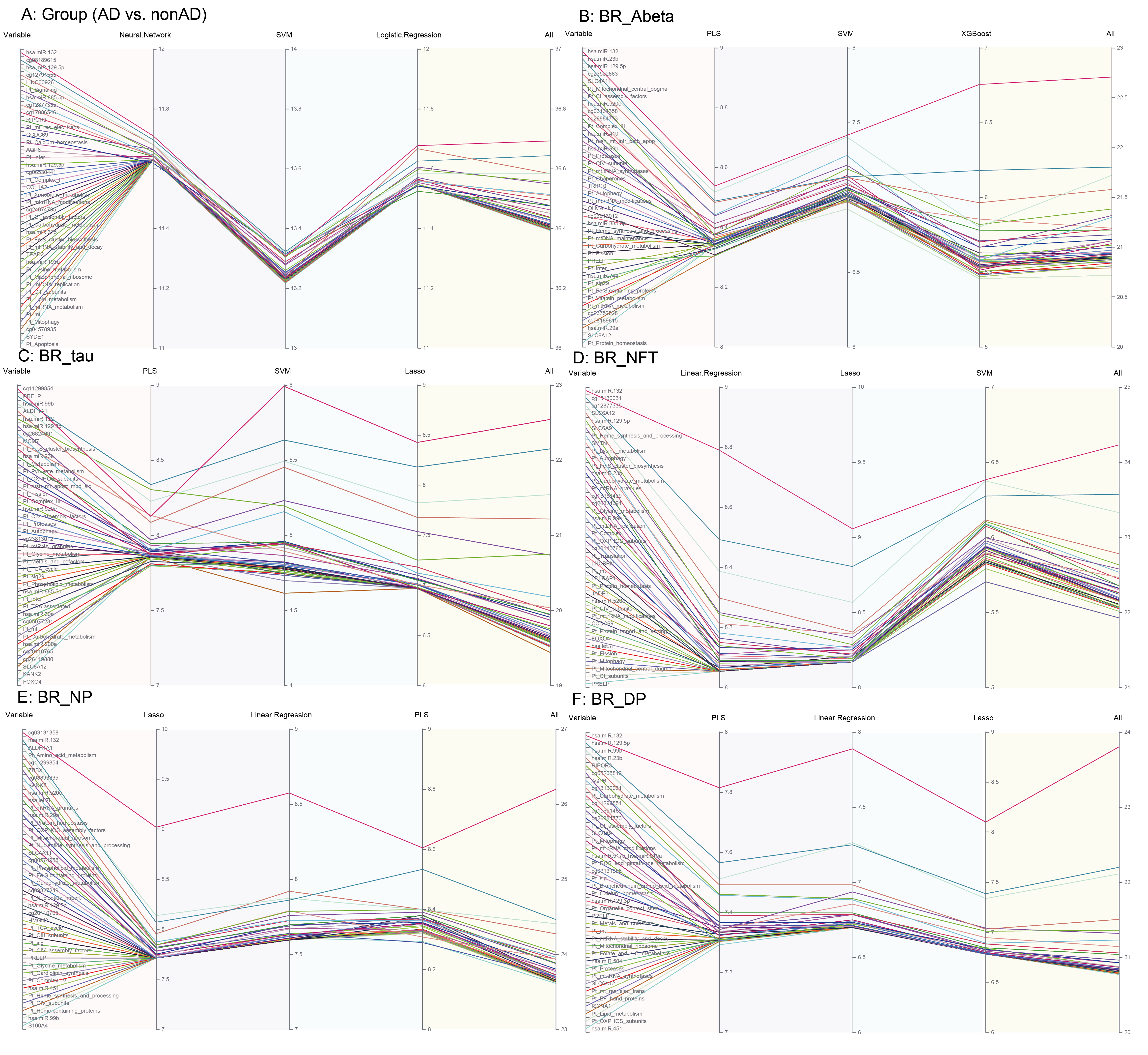


## Supplementary Fig.6 Integrative approach for identifying and validating important predictive features across multiple models and predictors.

To identify the most predictive features for the six predictors under investigation (one categorical and five regression metrics), a rigorous and integrative approach was employed. This approach involved strategic combination of the four types of most predictive characteristics derived from the top three models for each predictor, where each model was ranked in the upper 50% for model importance. The analytical workflow commenced with the meticulous ranking each model based on individual metrics, resulting in a composite ranking derived from the aggregate of these rankings. This methodical process of critical importance in determining the final hierarchy of models for each of the six predictive metrics. For each metric, the top three models were identified and selected based on their superior predictive efficiency. These models were then subjected to a detailed variable importance analysis. To investigate the variable importance of the models constructed within the “tidymodels” ecosystem, we leveraged the “DALEX” and “DALEXtra” R packages. These sophisticated packages provide sophisticated tools for enhancing model interpretability, enabling a deeper understanding of the models' behavior and the rationale behind their decision-making processes. The initial step involved the strategic division of the ROSMAP multi-omics data into training and test subsets, with a ratio of seven-to-three. Following the training of the model, the "DALEX" algorithm was employed to generate explainer objects. These objects were instrumental in deconstructing the models' predictions, facilitating a systematic assessment of the contribution of each predictor variable. Subsequently, the "DALEXtra" package was employed for its seamless integration with the tidymodels framework, enabling the efficient extraction and visualization of variable importance metrics. This integration not only streamlined our analytical workflow but also provided a comprehensive toolkit for elucidating the underlying mechanisms of our predictive models. By leveraging the capabilities of “DALEX” and “DALEXtra”, we were able to gain valuable insights into the relative importance of each predictor variable across the top three models for each predictive metric. This approach enabled the identification of the most influential features driving the predictive performance of our models, thereby providing a robust foundation for feature selection and model interpretation.


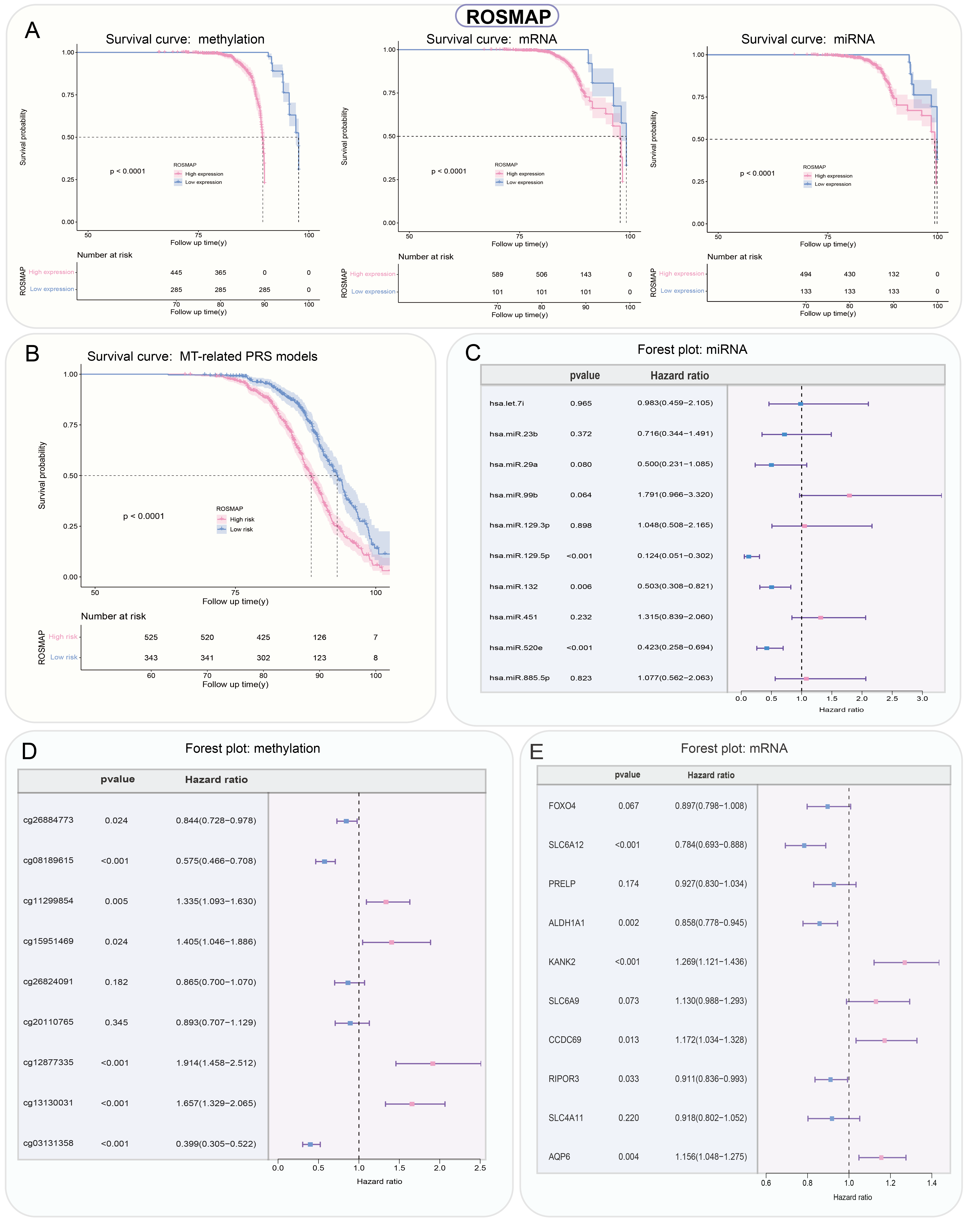


## Supplementary Fig.7 Survival and hazard ratio analysis of candidate biomarkers in the ROSMAP dataset reveals significant associations with AD risk across multiple omics layers.

(A-B) Our analysis of the ROSMAP dataset revealed that across four omics layers (genetic risk, miRNA, methylation, and mRNA), all features significantly distinguished between individuals at high and low risk for AD. Notably, those with higher expression levels experienced a significantly earlier onset of AD (*P* < 0.0001). This finding indicates the potential of these multi-omics features as predictive biomarkers for AD risk and progression. A multivariate Cox regression analysis was conducted to evaluate evaluated the association between these omics’ characteristics and AD risk. Our analysis identified eight MT pathway PRSs that surpassed our significance threshold of *P* < 0.05, suggesting their potential as genetic risk factors for AD. (C) At the miRNA level, three miRNAs—hsa-miR-129-5p, hsa-miR-520e, and hsa-miR-132—exhibited significant association with AD risk within the ROSMAP dataset. These miRNAs may play a crucial role in the regulation of gene expression and the pathophysiology of AD, making them promising candidates for further investigation as potential biomarkers or therapeutic targets. (D) Methylation data analysis pinpointed five sites (cg12877335, cg26824091, cg26884773, cg20110765, cg08189615) with significant AD risk correlation. These epigenetic modifications may contribute to the altered gene expression patterns observed in AD and provide insights into the underlying molecular mechanisms of the disease. (E) mRNA data analysis disclosed six features (*SLC6A12, KANK2, ALDH1A1, AQP6, CCDC69,* and *RIPOR3*) that exceeded the significance threshold, thereby reinforcing their potential as AD risk factors. These genes may be involved in various biological processes related to the AD pathogenesis, including neurotransmitter transport, cytoskeletal organization, and cellular metabolism.


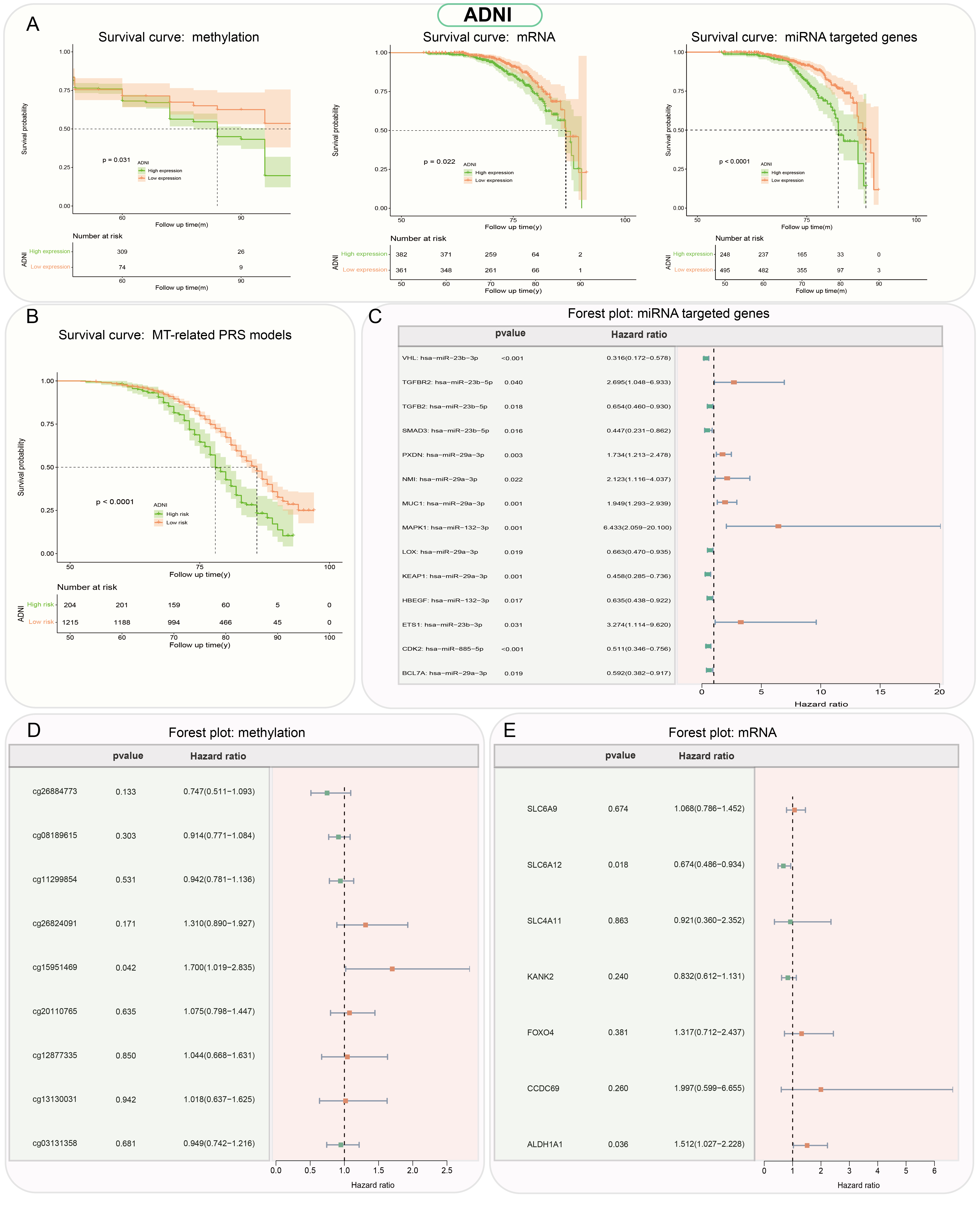


## Supplementary Fig.8 Validation of key biomarker associations in ADNI confirms and expands findings from ROSMAP.

(A-B) In the ADNI validation cohort, a profound distinction in AD risk was observed with the MT pathway PRSs and the miRNA targeted gene set, achieving highly significant statistical distinction (*P* < 0.0001). This finding corroborates the importance of mitochondrial genetic risk factors and miRNA-mediated regulation in AD pathogenesis, as identified in the ROSMAP dataset. Both methylation and mRNA profiles demonstrated notable discriminatory power for AD risk, with respective *P*-values of 0.031 and 0.022. This further supported the role of epigenetic modifications and transcriptomic alterations in AD susceptibility. (C) Upon translation to their target genes, five miRNAs—hsa-miR-23b-3p, hsa-miR-23b-5p, hsa-miR-29a-3p, hsa-miR-132-3p, and hsa-miR-885-5p—emerged as potentially significant contributors to AD risk in the ADNI dataset. Notably, hsa-miR-132 stood out as the sole miRNA validated for significance across both the ADNI and ROSMAP datasets. This consistent finding highlights the potential of hsa-miR-132 as a robust biomarker for AD risk and underscores its functional importance in the disease pathology. (D) In the ADNI dataset, only one methylation site (cg15951469) reached statistical significance. (E) For mRNA data, two features (*ALDH1A1* and *SLC6A12*) in the ADNI dataset surpassed the significance threshold, and were also identified as significant in the ROSMAP dataset. This consistent finding across two independent cohorts strengthens the evidence for the involvement of these genes in AD pathogenesis. Of particular interest is the significant negative correlation observed between *SLC6A12* and AD risk in both datasets (HR_ROSMAP_ = 0.784, HR_ADNI_ = 0.673). This suggests a potential protective association against AD risk.


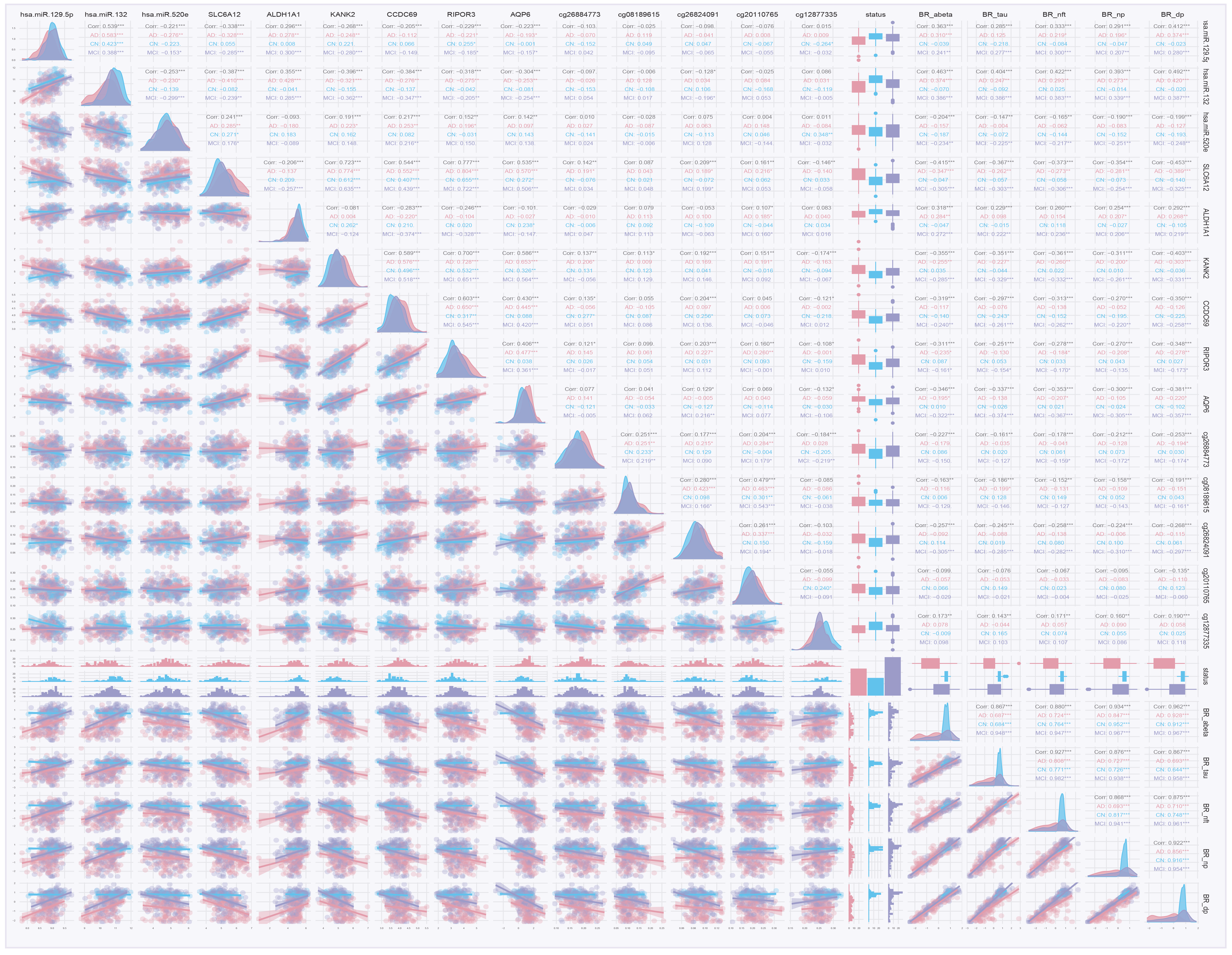


## Supplementary Fig.9 Correlation analysis in the ROSMAP reveals strong associations between candidate multi-omics features and their potential role in brain resilience.

In our comprehensive examination of the ROSMAP dataset for additional candidate features, we identified a highly significant positive correlation between *RIPOR3* and *SLC6A12*, with a correlation coefficient of 0.777 and a highly significant *P*-value of less than 0.001. This striking finding suggests a robust interplay between these two genes, which may play a critical role in the pathophysiology of AD. Furthermore, a significant positive correlation was identified between two microRNAs, hsa-miR-132 and hsa-miR-129-5p (Corr = 0.539, *P* < 0.001). This suggests a potential coordinated regulatory effect on shared target genes, which may have important implications for AD-related biological processes. Upon examination of the correlation between these miRNAs and brain resilience metrics, hsa-miR-132 exhibited the most significant positive associations, closely followed by hsa-miR-129-5p. These correlations suggest that these miRNAs may play a pivotal role in modulating the brain's capacity to withstand stress and degeneration in the context of AD pathology. In contrast, *SLC6A12* demonstrated the most pronounced negative correlations with all the metrics of brain resilience, indicating that higher expression levels of this gene may be inversely related to the brain's resilience to neurodegenerative processes.


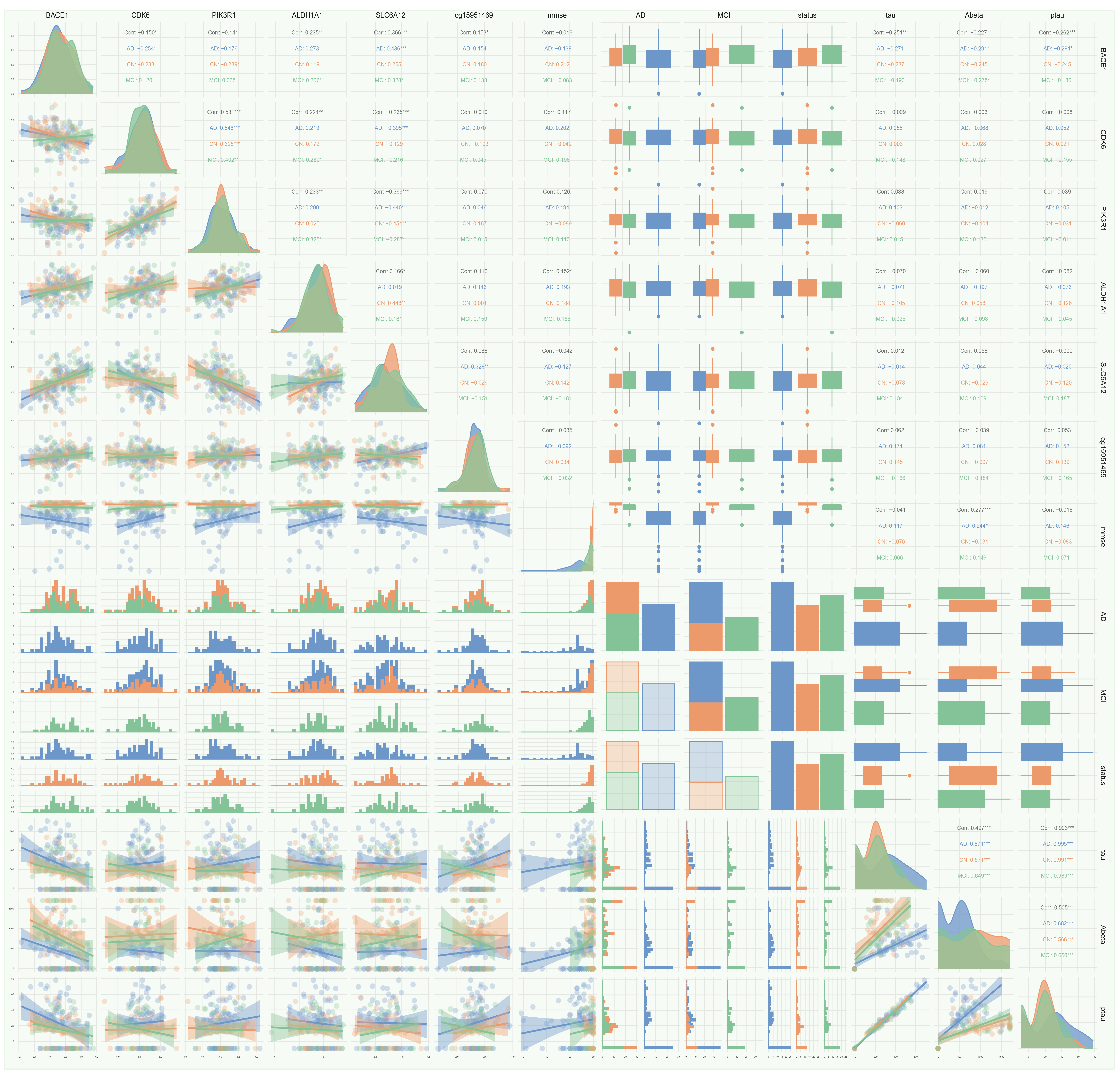


## Supplementary Fig.10 Correlation analysis in the ADNI dataset reveals potential features interactions and associations with AD pathology.

In the ADNI dataset, our analysis revealed the strongest positive correlation between *PIK3R1* and *CDK6* (Corr = 0.531, *P* < 0.001). This robust association suggests the potential for a synergistic relationship between these two genes in the context of AD. Conversely, *SLC6A12* demonstrated the strongest negative correlation with *PIK3R1* (Corr = -0.399, *P* < 0.001), suggesting a potential antagonistic interaction or inverse relationship with AD risk. Furthermore, *BACE1*, a key enzyme involved in the production of amyloid-beta peptides, exhibited the strongest negative correlations with the CSF indices: CSF-tau (Corr = -0.251, *P* < 0.001), CSF-ptau (Corr = -0.262, *P* < 0.001), and CSF-Abeta (Corr = -0.227, *P* < 0.01). These correlations provide further support for the involvement of *BACE1* in AD pathology and its association with well-established CSF biomarkers.


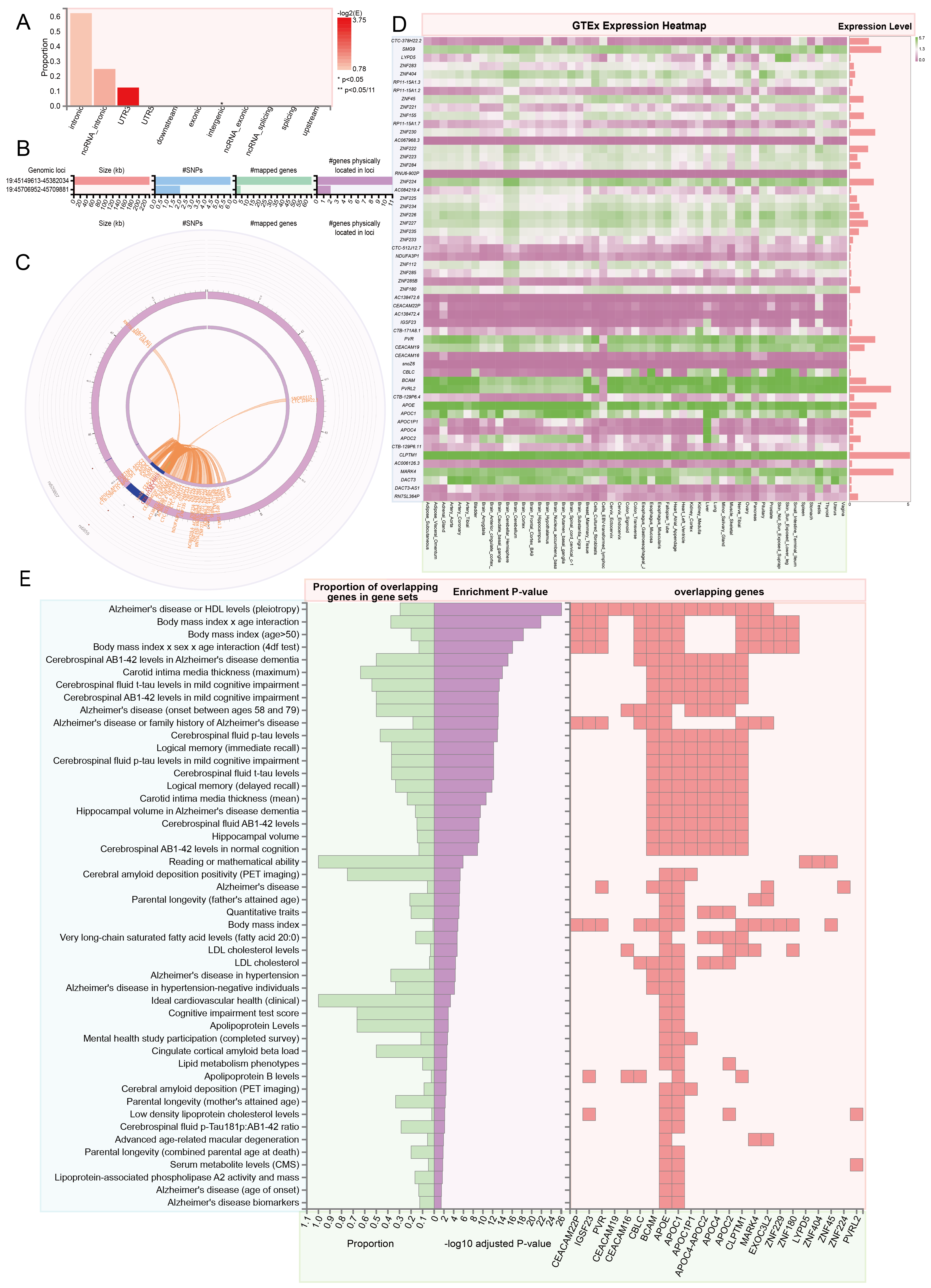


## Supplementary Fig.11 Comprehensive annotation of mitochondrial-related SNPs unveil their potential regulatory roles in AD pathogenesis.

(A) Our comprehensive genetic analysis identified two important PRS collections, comprising a total of 620 SNPs, predominantly located within intronic regions. The histogram depicted the distribution of SNPs that are in linkage disequilibrium (LD) with the independently significant (Ind.Sig) SNPs, which were functionally annotated using the ANNOVAR tool. The bars are shaded according to the logarithm (base 2) of the enrichment ratio, which compares the frequency of these annotated SNPs to those in the 1000 Genome Project reference panel phase 3. This annotation approach assigns biological context and potential impact to the genetic variants, thereby facilitating the interpretation of their functional significance. (B) Further annotation delineated two risk genomic regions on chromosome 19 (19:45149613-45382034 and 19:45706952-45709881) and pinpointed four lead SNPs: rs714948, rs57537848, rs6859, and rs620807. eQTL data highlighted the influence of these SNPs on the expression of *CEACAM19* across various brain tissues, suggesting their potential regulatory role in brain function and their relevance to AD pathogenesis. (C) Chromatin interaction and eQTL mapping revealed genes that were exclusively associated with the identified SNPs, which were highlighted in orange. The Hi-C interaction data (GSE87112) on chromosome 19 revealed significant connections between rs56261258 (*PVR*) and several genes, including ENSG00000216588 (*IGSF23*), ENSG00000159915 (*ZNF233*), and ENSG00000062370 (*ZNF112*). The findings indicate that the identified SNPs may exert their effects on AD risk through long-range chromatin interactions and the regulation of gene expression. (D) A comparative analysis of the identified SNPs against 68 genes revealed significant expression trends for *APOE, BCAM1, CLPTM1*, and *PVRL2* across 54 tissues in the GTEx project. This observation provides further support for the potential functional impact of these SNPs on gene expression and their possible involvement in AD-related biological processes. (E) Annotation of the GWAS catalog indicated significant enrichment of the identified genes and SNPs are significantly enriched in numerous AD and age-related processes, including body mass index age interaction (adj.*P* = 1.26×10^-22^) and CSF AD pathology levels, such as Aβ1-42, p-tau, and t-tau (adj.*P* = 4.34×10^-14^/6.33×10^-13^) (Table S17). Additionally, associations were observed between the genetic variants and behaviors related to memory and cognitive impairment, which serves to reinforce the validity of the analysis and the potential relevance of these genetic variants to AD pathogenesis.


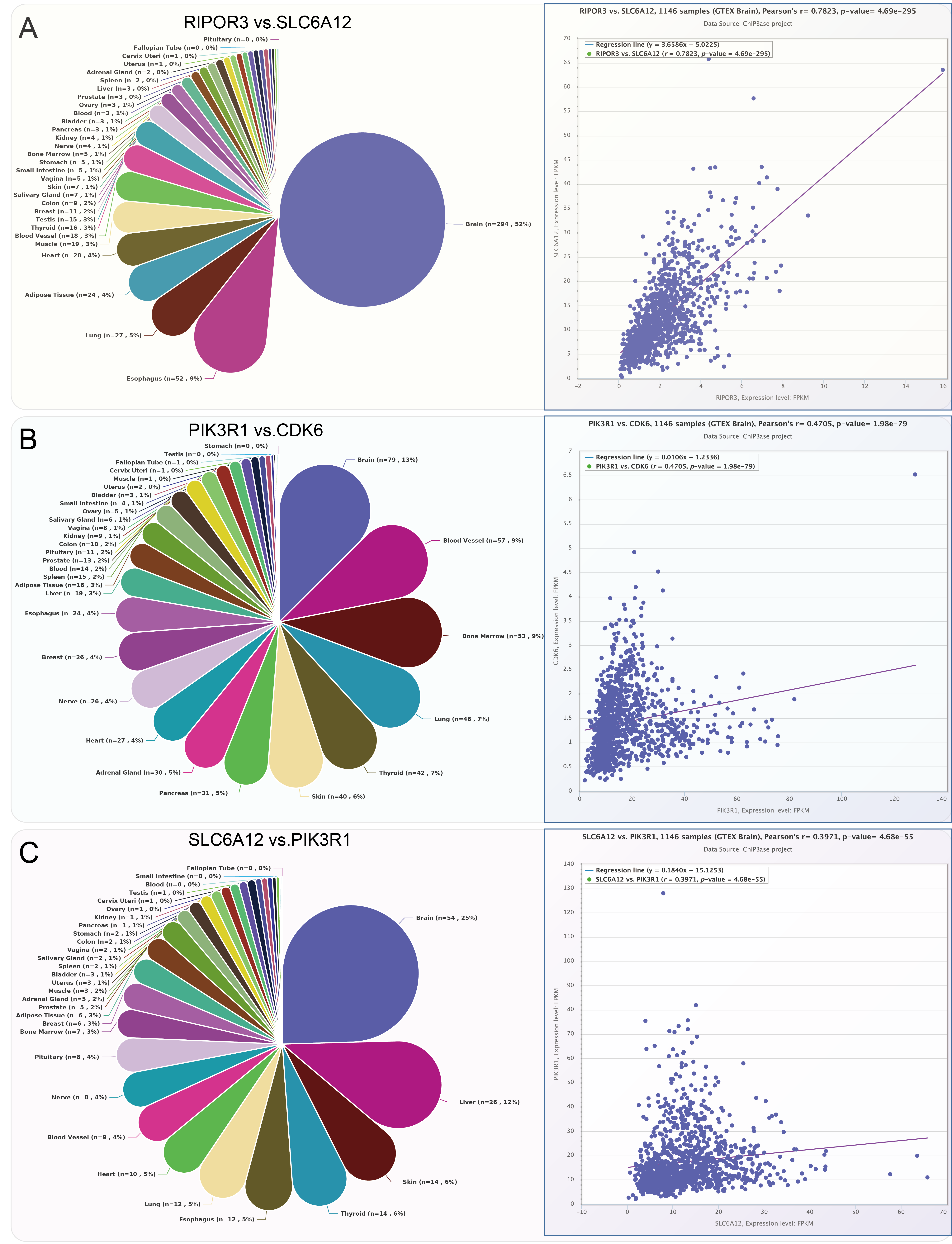


## Supplementary Fig.12 Tissue-specific co-expression analysis of candidate interacting genes reveals pronounced correlations in brain tissue.

(A) A comprehensive co-expression analysis of various tissues from the GTEx project, we identified the three most robustly correlated gene pairs: *RIPOR3* and *SLC6A12*, (B) *PIK3R1* and *CDK6*, and (C) the reciprocal pair of *SLC6A12* and *PIK3R1*. Remarkably, these gene pairs exhibited the most pronounced co-expression patterns in brain tissue, suggesting a potentially significant interplay within the neural context. This finding emphasizes the importance of these gene pairs in the brain's molecular landscape and may provide valuable insights into their collaborative roles in brain function and related pathologies. The identification of such robust correlations in brain tissue is particularly compelling, given the complex gene regulatory networks and interactions that underlie neurological processes. These findings underscore the necessity for further investigation into the functional consequences of these co-expression patterns and their potential implications for our understanding of brain function and disease.


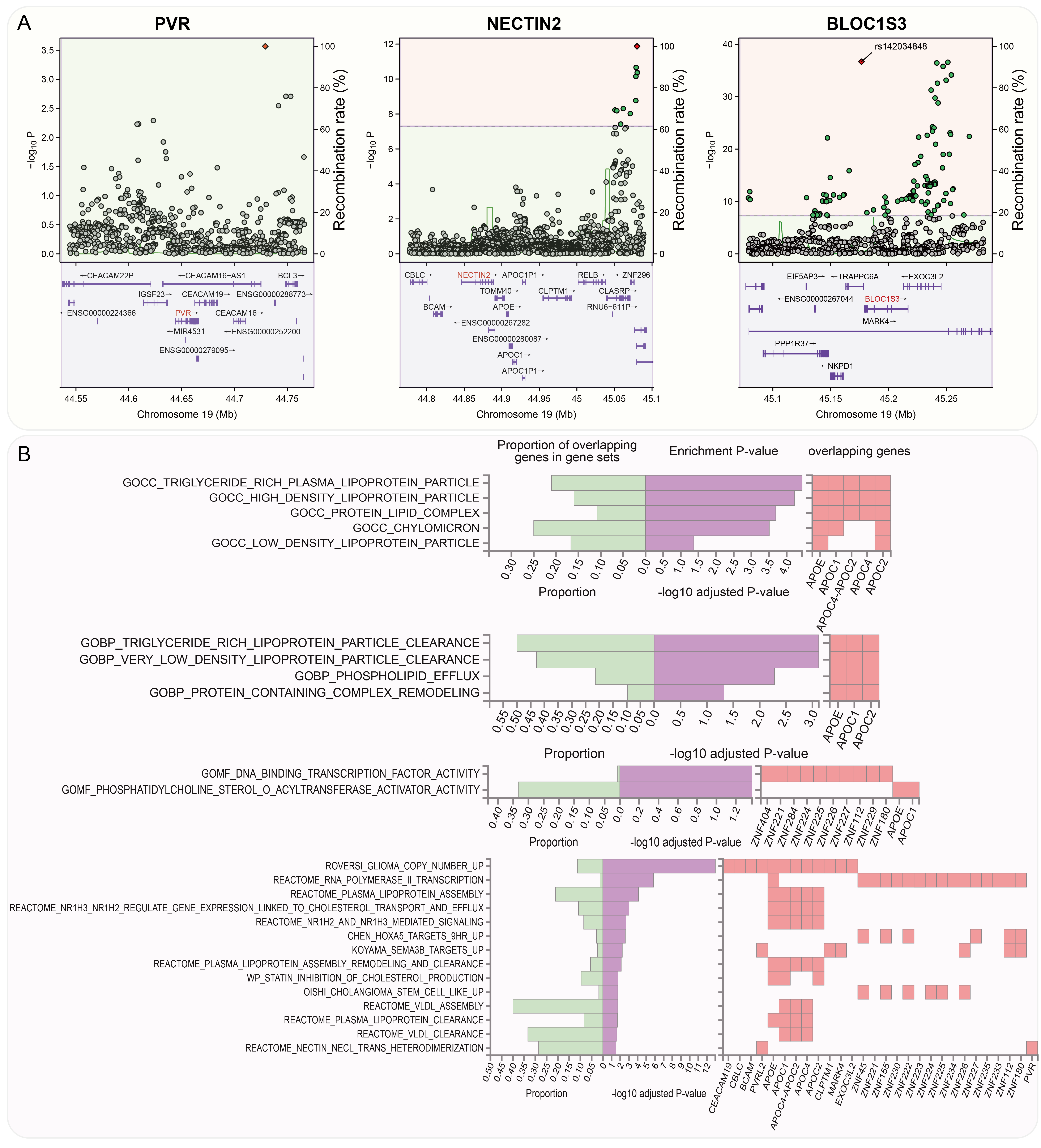


## Supplementary Fig.13 Mitochondrial-related biomarker landscape illustrates complex interactions and regulatory networks in AD pathogenesis.

(A) The comparative genomic landscape of mitochondrial-related biomarkers in AD is presented in a triptych format, with each plot illustrating the distribution and linkage disequilibrium of SNPs in proximity to the genes *PVR*, *NECTIN2*, and *BLOC1S3*, which have been implicated in AD pathogenesis. (B) The relationship between 68 mapped genes and lipoprotein particles is highlighted, with a particular focus on *APOE*, *APOC1*, *APOC2*, and *APOC4*. The regulatory role of zinc finger protein family genes, such as *ZNF404*, is underscored in conjunction with *APOE* and *APOC1*, influencing DNA-binding transcription factors and phospholipid-choline O-acyltransferase activity. This suggests a potential role in the regulation of HDL metabolism. eQTL analysis of brain tissues has revealed significant interactions between the identified SNPs and the mitochondrial epistatic gene *CEACAM19*. Furthermore, Reactome enrichment analysis has implicated *CEACAM19*, *PVR*, *BCAM*, *APOE*, and the aforementioned zinc finger proteins in plasma lipoprotein assembly, clearance, and the signal transduction pathways of *NR1H3* and *NR1H2*. Collectively, these insights unveil a unified regulatory impact of mitochondria, zinc finger proteins, and AD risk genes on the complex pathogenesis of AD.
